# Deep learning prediction of nocturnal hypertension for patients intolerant to ambulatory blood pressure monitoring

**DOI:** 10.1038/s43856-026-01639-x

**Published:** 2026-05-08

**Authors:** Yifan Lin, Mingwei Wen, Peiying Sun, Junjun Sun, Hao Yang, Yanfang Wang

**Affiliations:** 1 Department of Cardiology, The 1st Affiliated Hospital, Wannan Medical College, Wuhu, China; 2 Department of Biomedical Engineering, College of Life Science and Technology, Huazhong University of Science and Technology, Wuhan, China; 3 Department of Electronic Commerce, Anhui Business College, Wuhu, China; 4 Institute of Artificial Intelligence, Hefei Comprehensive National Science Center, Hefei, China

**Keywords:** Hypertension, Diagnosis

## Abstract

**Background:**

Ambulatory blood pressure monitoring (ABPM) plays an irreplaceable role in the diagnosis and management of hypertension. However, more than 100 million of the 1.4 billion people with hypertension worldwide cannot tolerate nighttime ABPM due to noise and arm compression. Previous prediction methods relying on demographic factors and home blood pressure measurements are time-consuming and burdensome while exhibiting limited accuracy for nocturnal hypertension. There is a need for a more accurate, low-burden approach to identify high-risk patients intolerant to nighttime ABPM monitoring.

**Methods:**

We collected 2,874 ABPM records at a regional medical center to conduct a retrospective cohort study. Kernel density estimation based preprocessing was applied to stabilize data fluctuations. A variational autoencoder based deep learning model was developed using daytime blood pressure and heart rate combined with full-day activity and posture states to predict nocturnal hypertension.

**Results:**

Here we show that the ABPM-VAE model achieves an AUC of 0.82 (95% CI 0.77-0.88) on the test set, outperforming the ablation model (AUC 0.67; 95% CI 0.61-0.74; *p* < 0.001) and prior methods based on demographic and home blood pressure data (AUC 0.69). For nocturnal hypertension prediction, the model yields a PPV of 92.12%, NPV of 55.20%, sensitivity of 0.73, and specificity of 0.84.

**Conclusions:**

The entropy reduction preprocessing-enhanced deep learning model predicts nocturnal hypertension risk from ABPM without adding burden to patients or physicians. It serves as an effective screening tool to identify high-risk individuals intolerant to nighttime monitoring, serving as a valuable complement to conventional ABPM.

## Plain language summary

ABPM remains the only validated method for diagnosing nocturnal hypertension. However, among the more than 1.4 billion people with hypertension, over 100 million cannot tolerate nighttime ABPM due to noise from cuff inflation and arm compression. Previous prediction approaches relying on demographic factors and home blood pressure measurements are time-consuming, burdensome, and offer limited accuracy. Using existing recorded ABPM data to predict nocturnal hypertension therefore represents a cost-effective and efficient alternative. By applying data smoothing to mitigate short-term fluctuations, we developed a deep learning method that improves the prediction of nocturnal hypertension compared with prior approaches. This method serves as a valuable complement to ABPM and enables effective screening of high-risk patients who are intolerant to nighttime ABPM.

## Abstract (web summary)

Lin et al. developed a Variational Autoencoder based deep learning model using daytime ambulatory blood pressure monitoring (ABPM) and activity/posture data to predict nocturnal hypertension. The study found that entropy-reduction preprocessing enhanced model performance, offering an effective supplement to ABPM for screening high-risk individuals intolerant to night monitoring.

## Introduction

The ABPM monitor is a wearable device and the only diagnostic method for nocturnal hypertension^1,2^. Despite its clinical value, 8.8% of patients cannot tolerate nighttime ABPM, and missed diagnoses of nocturnal hypertension increase the risk of severe cardiovascular diseases^3,4^. The J-HOP study involving 2765 patients attempted to predict nocturnal hypertension using demographic data and 72 blood pressure (BP) readings (three before and after sleep for 13 consecutive days), achieving an AUC of 0.69^5^. Previous methods are time-consuming, burdensome, and provide limited accuracy. By directly screening high-risk patients using ABPM data, the detection rate of nocturnal hypertension can be improved in intolerant patients without increasing the burden on healthcare providers or patients, thereby addressing an important unmet clinical need.

ABPM monitors record systolic and diastolic blood pressure (SBP/DBP), HR, activity, and posture status every half or one hour, providing clinicians with multidimensional data on patients’ BP status in real-life conditions^4^. Under real-life settings, BP and HR (BP-HR) exhibit variations in the high-frequency (0.15–0.40 Hz), low-frequency (0.04–0.15 Hz), and very-low-frequency bands (0.003–0.04 Hz) due to physiological factors such as baroreflex regulation and autonomic nervous system activity^6–11^. Within a 5-min interval, the standard deviations of SBP and DBP can reach 8.09 mmHg and 5.65 mmHg, respectively^12–14^. These fluctuations in BP-HR on a short time scale have hindered the clinical diagnosis reproducibility of circadian rhythm patterns, including Nondipping BP and isolated nocturnal hypertension^15–18^. Furthermore, posture and activity data-recorded as binary time series-affect BP-HR levels on both short and long time scales^19,20^. These records lack clinical context (e.g., indicators of insomnia or frailty), which complicates their transformation into low-dimensional, clinically meaningful continuous embedding vectors akin to ICD diagnostic codes^21–23^.

The BP fluctuations present significant challenges in predicting nocturnal hypertension, mainly because the sampling frequency does not meet the Nyquist criterion, which requires at least twice the maximum signal bandwidth^24,25^. As a result of aliasing, the short-time-scale fluctuations in BP-HR are folded into the circadian rhythms, which degrades the forecasting accuracy of time series models. Previous studies have shown that random perturbations can degrade forecast performances for exchange rates and power transformer temperatures by 27.26% and 81.06%, respectively^26^. Moreover, adversarial attack experiments on time series have demonstrated that even minor disturbances can significantly impair model prediction^27–32^.

Mitigating short-time-scale BP-HR fluctuations in ABPM helps reveal circadian rhythm characteristics and improves predictive model performance. However, cuff measurements taken at intervals shorter than 15 min compromise ABPM accuracy^33^, making it infeasible to simply increase the sampling frequency to reduce aliasing of high-frequency BP-HR components. This limitation also hinders the application of common denoising techniques, such as low-pass filtering and Empirical Mode Decomposition, which typically require high-frequency data acquisition^34–36^. In contrast, Kernel density estimation (KDE) does not rely on high-frequency data collection. By applying Gaussian kernel-based smoothing to every data point, KDE generates probability density values that attenuate short-time-scale fluctuations while preserving long-time-scale BP-HR characteristics. Unlike typical smoothing methods, which may reduce feature information, KDE enhances the effective dimensionality of the input data, thus avoiding significant information loss^37,38^.

In this study, we propose a preprocessing framework based on KDE combined with entropy reduction techniques, followed by a customized variational autoencoder (ABPM-VAE) model. This approach utilizes daytime BP-HR along with activity and posture data collected throughout the day that are transformed into continuous embedding vectors, to predict nocturnal hypertension. The method effectively attenuates short-time-scale fluctuations in BP-HR while preserving circadian rhythm characteristics and capturing nonlinear BP-HR interactions. The ABPM-VAE model achieves superior predictive performance compared with conventional approaches. Without increasing the burden on patients or healthcare providers, this approach facilitates the screening of high-risk patients who lack nocturnal recordings due to intolerance to nighttime ABPM, thereby addressing practical needs in clinical scenarios.

## Methods

### Data collection and study cohort

We collected 3531 ABPM records from the First Affiliated Hospital of Wannan Medical College between December 24, 2020, and March 20, 2023. ABPM measurements were performed using the DMS-ABP2 monitor, with readings taken every 30 min during the daytime (6:00 A.M. to 10:00 P.M.) and every hour during the nighttime (10:00 P.M. to 6:00 A.M.). Each reading included the measurement time, SBP, DBP, HR, activity status, and posture state. Of the collected records, 2920 had complete 24 h monitoring data and were initially retained. Among these, 42 patients contributed two records each and 2 patients contributed three records each. To ensure data independence, only the most recent ABPM record from patients with repeated measurements was included, resulting in a final cohort of 2874 independent patients (1492 men [51.9%] and 1382 women [48.1%]; mean [SD] age, 54.2 [15.7] years). Of these patients, 1592 (55.4%) had nocturnal systolic hypertension and 1703 (59.2%) had nocturnal diastolic hypertension. Nocturnal hypertension labels were defined as follows: systolic nocturnal hypertension was labeled positive (1) if the mean nighttime SBP (10:00 P.M.–6:00 A.M.) exceeded 120 mmHg and negative (0) otherwise; diastolic nocturnal hypertension was similarly defined using a mean nighttime DBP threshold of 70 mmHg. Demographic and clinical data, including gender, age, medical history, medication usage over the past three months, most recent laboratory results, and echocardiography parameters, were extracted from the outpatient and inpatient electronic medical record systems. Key cohort characteristics are summarized in Supplementary Table S1 and S2. The study adhered to the principles outlined in the Declaration of Helsinki and received approval from the Scientific research IRB of Wannan Medical College Yijishan Hospital. All data were retrieved from the hospital’s electronic medical record system. Given the retrospective design, exclusive use of fully anonymized existing clinical data (anonymized during extraction using unique identifiers from the electronic medical record system, followed by random reassignment of new identifiers), and no more than minimal risk to participants, the requirement for informed consent was waived.

### Dataset splitting and subset generation

The dataset was randomly split at the patient level in a 9:1 ratio, with 90% (2584) of patients assigned to training and validation sets and the remaining 10% (290) to an independent test set. For patients in the training and validation sets, one reading per hour was randomly selected from the daytime interval (6:00 A.M. to 10:00 P.M.), yielding 16 daytime readings per patient. These were combined with the 8 nighttime readings to construct a complete 24-hour ABPM subset. For each original recording, ten such subsets were generated: eight assigned to the training set and two to the validation set. The same subset generation strategy was applied to the test set. This approach allowed assessment of model generalization on data from previously unseen patients. Since training and validation subsets originated from the same original recordings, strong performance on both with poor test set performance indicate overfitting to individual-level noise, whereas consistently poor performance across validation and test sets suggests failure to capture subject-specific physiological patterns.

### Entropy reduction preprocessing for blood pressure and heart rate

**Fig. 1:**
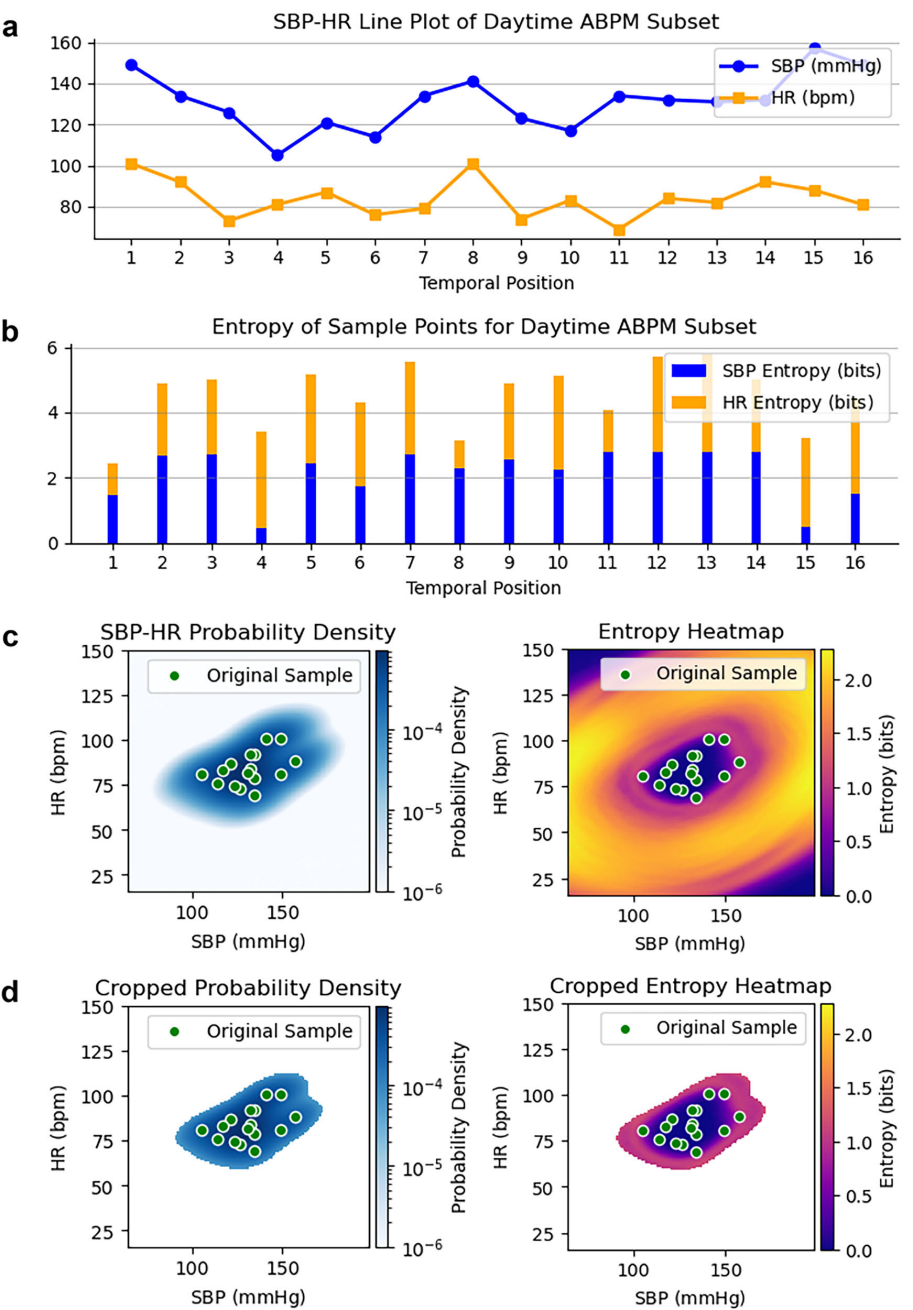
Entropy of SBP-HR data point bin assignments before and after entropy reduction preprocessing. **a** Line plot for the Daytime ABPM subset (ID: 1620#2021-03-20). The blue line represents SBP recorded between 6:00 AM and 10:00 PM, and the yellow line depicts HR over the same time. **b** Bar plot showing entropy of sample points in the ABPM subset. Blue bars: SBP; Yellow bars: HR. **c** Left: KDE-based probability density plot of SBP-HR; darker colors indicate higher density. Right: Corresponding entropy heatmap from Monte Carlo simulation using 10-bin method; darker colors indicate lower entropy. Green points show original data. **d** Left: Cropped KDE density plot excluding grid points with density below 5% of maximum. Right: Corresponding entropy heatmap. Green points show original SBP-HR data points.

We applied KDE to estimate the probability density distributions of SBP and HR, as well as DBP and HR, thereby generating BP-HR probability density distributions. These distributions were mapped onto a 256 × 256 grid to create corresponding probability density maps (Fig. 1c and Supplementary Fig. 1c). Subsequently, grid points with values below 5% of the maximum in the density map were set to zero (Fig. 1d and Supplementary Fig. 1d). Finally, the entire density map was weighted by dividing its values by the square root of its maximum value. KDE is a widely used non-parametric method that does not assume a specific distribution for the data. Instead, it constructs a density estimate by smoothing data points, making it particularly suitable for estimating unknown probability density functions from a given sample set.

The KDE is defined as follows. Given a set of samples for SBP *X* = {*x*_1_, *x*_2_, …, *x*_*n*_} and HR *Y* = {*y*_1_, *y*_2_, …, *y*_*n*_}, the joint probability density function *p*(*x*, *y*) using a Gaussian kernel is expressed as:

$$p(x,y)=\frac{1}{n}{\sum }_{i=1}^{n}\frac{1}{2\pi {h}^{2}\sqrt{| \Sigma | }}\exp \left(-\frac{1}{2{h}^{2}}{\left[\begin{array}{c}x-{x}_{i}\\ y-{y}_{i}\end{array}\right]}^{T}{\Sigma }^{-1}\left[\begin{array}{c}x-{x}_{i}\\ y-{y}_{i}\end{array}\right]\right)$$

where, *p*(*x*, *y*) denotes the joint probability density of *X* and *Y*, *n* is the number of samples, *Σ* is the covariance matrix of the samples, and *Σ*^−1^ is its inverse, which reflects the covariance relationships between the variables. The parameter *h* is the bandwidth or smoothing parameter, and $\exp$ denotes the exponential function.

In this study, Scott’s Rule was applied to determine the bandwidth *h*, defined as:

$$h={n}^{-1/(d+4)}$$

The covariance matrix *Σ* is calculated as:

$$\varSigma =\left[\begin{array}{cc}\frac{1}{n-1}{\sum }_{i=1}^{n}{({x}_{i}-\bar{x})}^{2} & \frac{1}{n-1}{\sum }_{i=1}^{n}({x}_{i}-\bar{x})({y}_{i}-\bar{y})\\ \frac{1}{n-1}{\sum }_{i=1}^{n}({x}_{i}-\bar{x})({y}_{i}-\bar{y}) & \frac{1}{n-1}{\sum }_{i=1}^{n}{({y}_{i}-\bar{y})}^{2}\end{array}\right]$$

### Evaluating the uncertainty in the numerical ordering of measurement points

ABPM recordings of BP-HR measurements are sampled from a distribution within a certain range, with significant short-time-scale fluctuations causing aliasing, superimposed on relatively stable long-term fluctuations. To evaluate the impact of preprocessing, we assessed the consistency of the numerical ordering of data points before and after preprocessing by calculating the entropy of their bin assignments. Assuming that each measurement follows a normal distribution *N*(*μ*, *σ*^2^), where *μ* represents the value at each ABPM measurement point and *σ* is the empirically determined standard deviation (see Supplementary Note 3), we used a Monte Carlo simulation to generate 1,000 BP-HR sequences for each ABPM subset along with their corresponding probability density maps. Specifically, for every measurement point in each sample, simulated values were drawn from the normal distribution *N*(*μ*, *σ*^2^), and the simulated BP-HR sequences were subsequently processed using KDE to obtain the probability density maps. In every simulated sequence or probability density map, data points were discretized into 10 bins based on the numerical ordering of the data points, with bin boundaries defined by the corresponding percentiles. After 1000 simulations, each data point in every sequence or probability density map was assigned 1000 corresponding bin labels. The entropy of these labels was then calculated, with lower entropy indicating higher consistency. Thus, entropy quantifies the stability of the data points derived from the same distribution before and after preprocessing. The discretized entropy *H*(*X*) for the bin labels at each measurement point is defined as:

$$H(X)=-{\sum }_{i=1}^{10}p({x}_{i})\log p({x}_{i})$$

where *p*(*x*_*i*_) denotes the probability of a measurement falling within the *i*-th bin.

### Cluster analysis of activity and posture transition probability based on statistical metrics

**Fig. 2:**
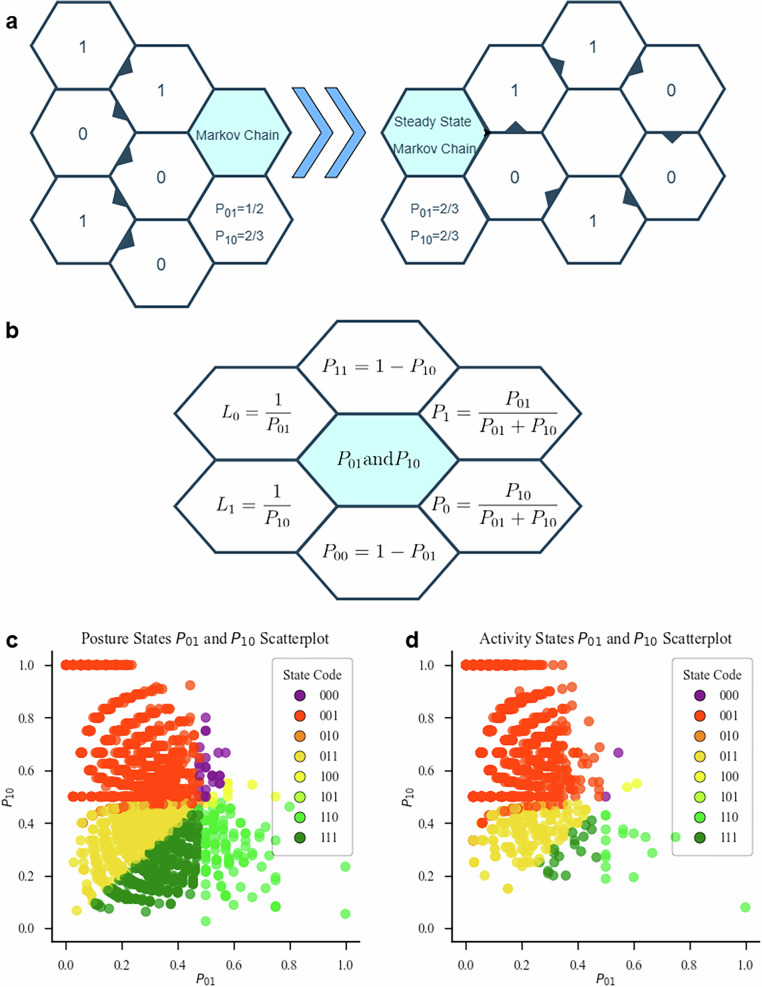
Activity and posture transition probabilities as low-dimensional embeddings show clustering by statistical metrics. **a** The left panel illustrates the Markov chain for the example sequence “110,010,” where black triangular arrows indicate temporal progression. When these transitions are rearranged into a cyclically repeating pattern, as shown in the right panel, the Markov chain reaches its steady-state distribution. **b** Once the two-state Markov chain reaches steady state, the transition probabilities *P*_01_ and *P*_10_ can be used to derive *P*_11_, *P*_00_, the proportions of “1''s (*P*_1_) and “0''s (*P*_0_), and their respective average run lengths (*L*_1_ and *L*_0_). In effect, the two transition probabilities encapsulate six statistical metrics. Here, *P*_01_ denotes the probability of transitioning to state 1 given that the current state is 0, and *P*_10_ denotes the probability of transitioning to state 0 given that the current state is 1. **c** The left panel presents the two-dimensional distribution of *P*_01_-*P*_10_ for all samples' posture time series. Each sample is assigned a three-digit binary state code based on the following criteria: the first digit is set to 1 if the sequence contains more than 50% “1''s; the second digit is 1 if the average run length of “1''s exceeds 2; the third digit is 1 if the average run length of “0''s exceeds 2. *n* = 2874 biologically independent patient samples. **d** The right panel shows the corresponding *P*_01_-*P*_10_ distribution for activity time series, using the same state code assignment criteria as in (c). *n* = 2874 biologically independent patient samples. We then applied linear discriminant analysis (LDA) to the *P*_01_-*P*_10_ scatter plot. LDA results show excellent separation by simple straight lines, with classification accuracies of 90.15% (posture) and 96.35% (activity), and separation ratios of 4.198 (posture) and 5.357 (activity), indicating very clear decision boundaries.

We denote “0” for resting or supine, and “1” for active or standing. These activity and posture time series form a Markov chain, where the transition probabilities P01 and P10 represent the probability of transitioning to state 1 given the current state is 0, and the probability of transitioning to state 0 given the current state is 1, respectively. Taking the sequence “110,010” as an example, when a sequence such as “110,010” is treated as a cyclically repeating pattern, the sequence reaches the steady-state distribution of the Markov chain (Fig. 2a). In this state, P01 and P10 can be used to directly derive P11, P00, the steady-state proportions P1 and P0, and their average consecutive lengths (Fig. 2b).

We further analyzed the clustering patterns in the P01-P10 scatter plots across all samples, using statistical metrics as class labels. The statistical metrics were represented using a three-digit binary code and marked with different colors: the first digit was set to 1 if the proportion of state 1 exceeded 50%; the second digit was set to 1 if the average run length of state 1 exceeded 2; and the third digit was set to 1 if the average run length of state 0 exceeded 2; otherwise, it was set to 0.

We then applied linear discriminant analysis (LDA) to evaluate the separability of samples labeled with different statistical metrics codes in the P01-P10 scatter plot, reporting classification accuracy and separation ratio (between-class distance/within-class standard deviation).

### ABPM-VAE and ablation models

**Fig. 3:**
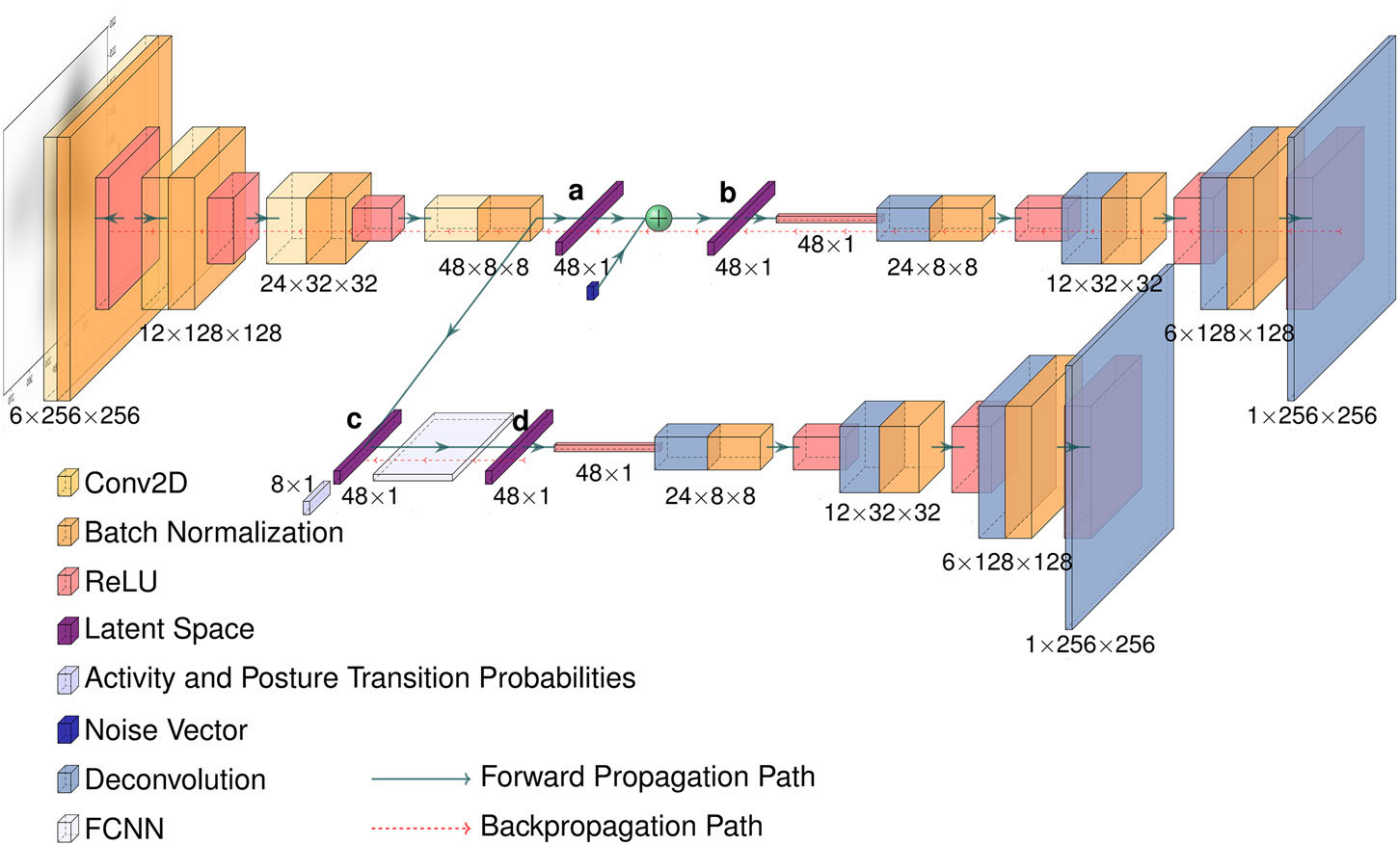
ABPM-VAE Architecture and Data Flow. **T**he ABPM-VAE model consists of two interconnected pathways that jointly predict nocturnal BP-HR probability density maps from preprocessed ABPM data. Upper pathway (reconstruction branch): The input BP-HR probability density map is passed through multiple convolutional layers to extract features and generate latent space a (purple bar a). A noise vector is then integrated to enhance continuity within the latent space, producing latent space b (purple bar b). Subsequently, deconvolution layers upsample to restore the spatial dimensions. The output of the upper pathway is trained to match the original input BP-HR probability density map (labels = input BP-HR probability density map), ensuring a compressed latent representation is achieved through reconstruction. All parameters in this pathway are optimized via backpropagation. Lower pathway (prediction branch): The daytime BP-HR probability density map is processed using an identical convolutional structure as the upper pathway to extract features and generate latent space c (purple bar c). This latent space c is then concatenated with the full-day Activity and Posture Transition Probabilities (*P*_01_-*P*_10_) and fed into a FCNN to learn the transformation from daytime to nighttime latent features, producing predicted latent space d (purple bar d). The resulting nighttime latent features are upsampled via deconvolution layers (with the same parameters as those in the upper pathway) to generate the predicted nocturnal BP-HR probability density map. The predicted map is compared against the true nocturnal BP-HR probability density map (labels = real nocturnal BP-HR probability density map). In the lower pathway, the deconvolution layer parameters are fixed (shared with the upper pathway), and backpropagation is applied exclusively to the FCNN parameters.

In a typical VAE, input data are encoded into a latent probabilistic distribution, which is then decoded to reconstruct the original input, learning low-dimensional representations^39–41^. Our ABPM-VAE model consists of two interconnected pathways (Fig. 3). The upper pathway compresses and reconstructs the input BP-HR probability density maps to form a latent space representation. Specifically, the input data is passed through multiple convolutional layers to extract features and create the latent representation. A noise vector sampled from *N*(0, 1) is then integrated to enhance continuity within this latent space, as preliminary experiments showed that the VAE reparameterization coefficients *u* approximate 0 and *σ* approximate 1. Subsequently, deconvolution layers restore the spatial dimensions. The output of the upper pathway is trained to match the original input map, ensuring that a compressed latent representation is achieved through reconstruction. The total loss function consists of two components: (1) the reconstruction loss (MSE between the output density map and the input density map); and (2) the L2 regularization on *u* (where *u* is the latent space vector). All parameters in this pathway are optimized via backpropagation.

The lower pathway uses the latent representation of daytime BP-HR probability density maps combined with transition probabilities of activity and positional states as input to a fully connected neural network (FCNN) to learn the transformation of the latent features of nighttime hypertension. The nighttime hypertension latent features are then upsampled via deconvolution to generate the predicted nighttime BP-HR probability density maps. ABPM-VAE outputs nocturnal SBP-HR and DBP-HR joint density maps. Marginalization over HR yields SBP/DBP marginal densities, whose means are predicted nocturnal BP. Nocturnal SBP labels are positive (1) if predictedSBP 120 mmHg (else 0); Nocturnal DBP labels are positive (1) if predicted DBP 70 mmHg (else 0). The lower pathway is supervised by a reconstruction loss, calculated as the MSE between the predicted and actual nighttime density maps. The convolution and deconvolution layers in the lower pathway are set to the same parameters as those in the upper pathway, and backpropagation is applied exclusively to the FCNN parameters in the lower pathway.

In the ablation model, we constructed a FCNN using raw ABPM data to predict continuous nocturnal SBP and DBP values (see Supplementary Note 4). The FCNN model takes as input the timestamp, SBP, DBP, HR, activity state, positional state, and a binary missingness indicator (1 for daytime measurements and 0 for withheld nighttime values). The output labels are the average nocturnal SBP and DBP, and nocturnal hypertension labels were then derived from FCNN outputs using the same clinical thresholds as the ABPM-VAE model (SBP 120 mmHg or DBP 70 mmHg for positive labels). The model is trained using MSE as the loss function between outputs and true nocturnal SBP/DBP labels. All parameters were optimized via backpropagation.

Since the convolutional and deconvolutional modules only compress and reconstruct the input probability density maps, ABPM-VAE and ablation model both rely on the FCNN to extract features indicative of nocturnal hypertension. The key distinction is that the ABPM-VAE model receives inputs processed through the entropy reduction preprocessing. Therefore, a comparison of their performance on the ABPM dataset serves to validate the effectiveness of the entropy reduction preprocessing.

### Baseline model comparisons

To benchmark the proposed ABPM-VAE framework, three established baseline models were implemented for predicting nocturnal SBP/DBP from daytime ABPM time-series data: Light Gradient Boosting Machine (LightGBM) and Long Short-Term Memory (LSTM) models. LightGBM, a state-of-the-art decision tree model, is widely recognized as a leading method for time series analysis, as evidenced by its exceptional performance in the M5 competition^42–44^. Input samples were represented as 168-dimensional feature vectors encompassing timestamp, SBP, DBP, HR, activity, posture and missingness indicator information. The LSTM model was selected as a representative classical recurrent neural network architecture widely used in healthcare sequential prediction tasks^45–47^. Each input sample consisted of 24 time steps with 7 features per step (timestamp, SBP, DBP, HR, activity, posture and missingness indicator), reshaped from a 168-dimensional feature vector with nocturnal SBP and DBP values masked to zero. All baseline models predicted continuous nocturnal SBP/DBP values. The loss function for training and performance evaluation across training, validation, and test sets were based on MSE.

### Additional phenotype prediction

To extend the evaluation of the ABPM-VAE model beyond nocturnal hypertension, additional analyses were performed for dipping pattern classification and BP variability assessment.

Dipping pattern classification used the same predicted mean nocturnal SBP from ABPM-VAE outputs as for nocturnal hypertension prediction. Non-dipping status was defined as a nocturnal SBP fall < 10% relative to the patient’s daytime mean SBP. Since both classifications derive from identical predicted nocturnal BP values, we assessed sensitivity to prediction error for dipping patterns and nocturnal hypertension. Noise-sensitivity simulations added random signed Gaussian noise *ε* = S × ∣*N*(0, *σ*)∣(S uniformly sampled from {−1, +1}) to reference nocturnal BP means, with *σ* ranging from 1 to 20 mmHg; 1000 Monte Carlo iterations were performed per noise level and patient, evaluating classification consistency accuracy and AUC for both dipping patterns and nocturnal hypertension across noise intensities.

BP variability was quantified using the coefficient of variation (CV) computed from the model’s predicted nocturnal BP-HR joint probability density maps. For each map, marginalization over the HR axis yielded the marginal SBP/DBP distribution. The weighted mean and weighted standard deviation were calculated using this marginal distribution as weights, and CV was defined as weighted standard deviation divided by weighted mean. We analytically derived a proportional scaling relationship between CV computed from the probability density map (CV(map)) and from raw measurements (CV(sample)). This conclusion was empirically validated using Bland-Altman analysis. The scaling coefficient *k* was determined via least-squares fitting. CV(sample) was obtained as *k* × CV(map) for variability estimation.

### Model interpretability analyses

To enhance the interpretability of the ABPM-VAE model, two analyses were performed: vanilla gradient saliency mapping and targeted perturbation experiments.

Vanilla Gradient saliency maps were generated for the ABPM-VAE model to assess feature importance. Gradients were backpropagated with respect to the input daytime BP-HR probability density maps. Absolute gradient values were taken and normalized across the input space for visualization, with high-saliency regions indicating areas of strong model attention. This approach identified the input features most influential in the model’s predictions of nocturnal BP-HR probability density maps.

Targeted perturbation experiments were conducted to further elucidate the influence of activity and posture transition probabilities on the model’s predictions of nocturnal BP by designing four extreme scenarios: 1: Daytime activity and posture transition probabilities (P01, P10) set to [0, 1, 0, 1]; nighttime unchanged; 2: Nighttime activity and posture transition probabilities set to [0, 1, 0, 1]; daytime unchanged. In Scenarios 1 and 2, this configuration simulates a patient remaining in a resting/supine state (P01 = 0, P10 = 1); 3: Daytime activity and posture transition probabilities set to [1, 0, 1, 0]; nighttime unchanged; 4: Nighttime activity and posture transition probabilities set to [1, 0, 1, 0]; daytime unchanged. In Scenarios 3 and 4, this simulates the opposite: a patient remaining in an active/standing state (P01 = 1, P10 = 0). For each scenario applied to all test samples, the mean differences in predicted nocturnal systolic and diastolic BP (perturbed minus original prediction; mean ± SEM) were calculated, and directional impacts were statistically assessed using two-tailed paired *t*-tests.

### Statistics and reproducibility

We quantified the uncertainty in the numerical ordering of measurement points before and after preprocessing by computing the discretized entropy on both randomly selected samples and the entire dataset. All data point classifications in P01–P10 scatter plots were displayed, reporting accuracy and separation ratio under different threshold conditions. Predictive accuracy of FCNN and ABPM-VAE models was evaluated on ABPM subsets from training, validation, and test sets across 1–200 training epochs. ROC analysis was performed for nocturnal hypertension prediction, computing AUC with 95% confidence intervals via 1000 bootstrap resamples, along with PPV, NPV, sensitivity, and specificity. Consistency of nocturnal hypertension predictions for FCNN and ABPM-VAE was compared under different within-hour selection strategiesusing Cohen’s Kappa analysis. Baseline model comparisons (LightGBM, LSTM) versus ABPM-VAE and ablation models used MSE, with two-tailed *t*-test for *p*-values. LightGBM hyperparameters were systematically varied (max_depth: 3-5; n_estimators: 100-1000; num_leaves: 8-128) to ensure comprehensive coverage. LSTM results provided MSE across 1-200 epochs for training, validation, and test sets. For additional phenotype prediction, noise-sensitivity for dipping patterns and nocturnal hypertension used 1000 Monte Carlo iterations per noise level and patient. BP variability’s proportional scaling was analytically derived and validated via Bland-Altman analysis. Vanilla Gradient saliency maps were generated deterministically by backpropagating gradients with respect to input density maps, taking absolute values, and normalizing for visualization. Data preprocessing and analysis were implemented in Python 3.8.0 with the statistics module; FCNN and ABPM-VAE models in PyTorch 1.13.0 with CUDA 11.6 support.

## Results

### Entropy reduction preprocessing provides a stable representation of BP-HR distributions

The BP-HR time series data from a randomly selected patient (Fig. 1a and Supplementary Fig. 1a) yielded average entropy values of 2.27 bits (SD 0.72 bits) for HR-SBP and 2.29 bits (SD = 0.74 bits) initially (Fig. 1b and Supplementary Fig. 1b). Subsequently, we applied a non-parametric Gaussian KDE approach to smooth the SBP-HR and DBP-HR data, thereby generating probability density plots (Fig. 1c and Supplementary Fig. 1c) where the average entropy values significantly decreased to 1.50 bits (SD 0.62 bits) for HR-SBP and 1.46 bits (SD 0.56 bits) for HR-DBP (*p* < 0.001, one-tailed *t*-test). Notably, regions with lower probability density exhibited higher entropy values. By excluding data points with probability densities below 5% of the maximum, we further reduced data uncertainty while retaining essential distribution information. Consequently, the values of cropped probability density plots (Fig. 1d and Supplementary Fig. 1d) significantly decreased to 0.65 bits (SD 0.46 bits) for HR-SBP and 0.46 bits (SD 0.47 bits) for HR-DBP(*p* < 0.001, one-tailed *t*-test).

**Fig. 4:**
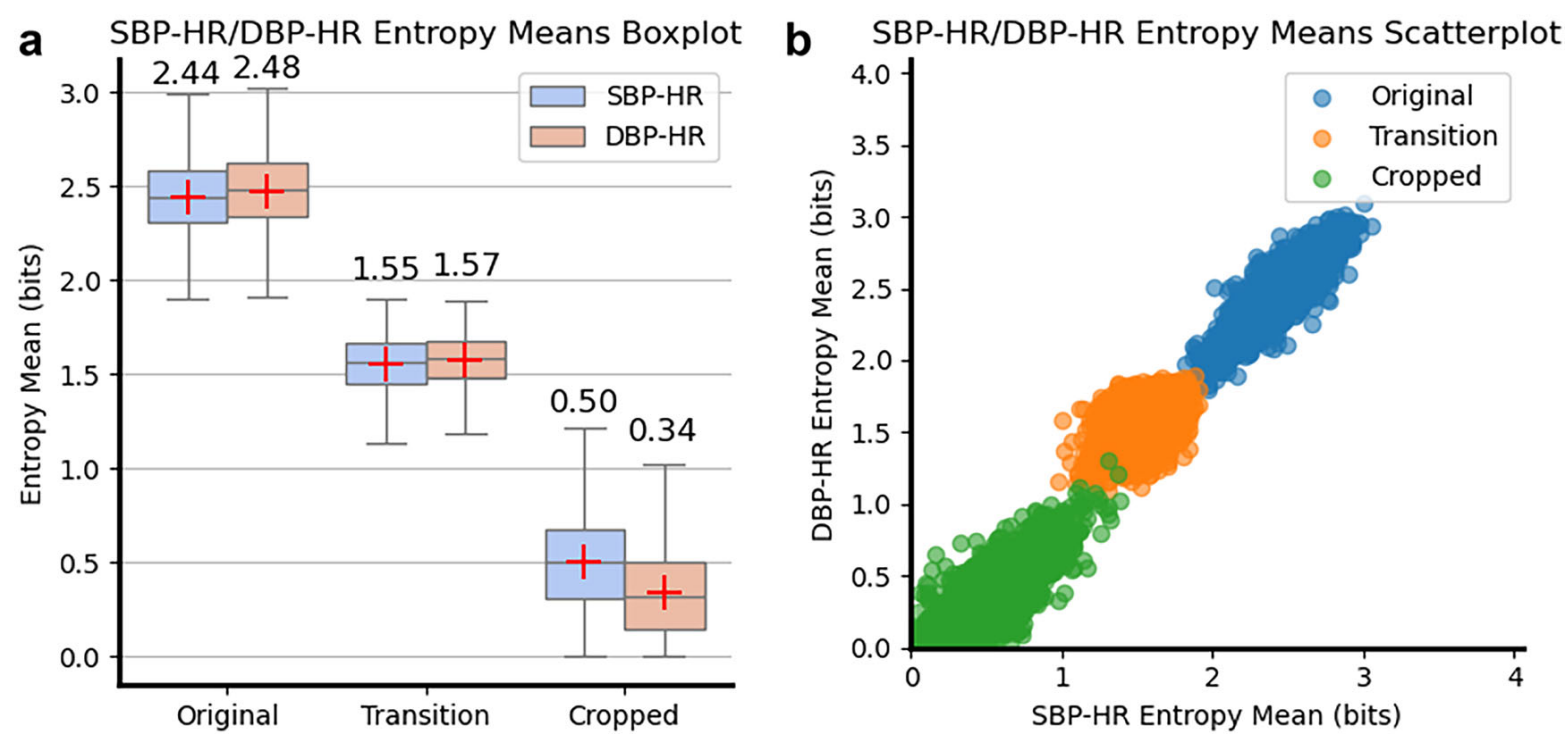
Entropy of overall sample data point bin assignments before and after entropy reduction preprocessing. **a** Box plots showing the changes in data point entropy for SBP-HR and DBP-HR before and after preprocessing. Blue boxes represent SBP-HR and orange boxes represent DBP-HR. Within each box, the central line indicates the median, and the red plus sign marks the mean. The box edges correspond to the first and third quartiles, while the whiskers extend to ±1.5 times the interquartile range, *n* = 2874 biologically independent patient samples. The original average entropy values are 2.44 bits (SD 0.20 bits) for HR-SBP and 2.48 bits (SD 0.21 bits) for HR-DBP; the transition average entropy values signiffcantly decrease to 1.55 bits (SD 0.15 bits) and 1.57 bits (SD 0.14 bits), respectively (*p* < 0.001, one-tailed *t*-test); the Cropped entropy average entropy values signiffcantly decrease to 0.50 bits (SD 0.25 bits) for HR-SBP and 0.34 bits (SD 0.23 bits) for HR-DBP (p < 0.001, one-tailed *t*-test). **b** Scatter plots illustrating the two-dimensional distribution of data point entropy for SBP-HR and DBP-HR across three stages: “original” denotes the raw time series; “transition” represents the KDE-based probability density plots; and “cropped” indicates the density plots after excluding grid points with probability densities below 5% of the maximum value.

Figure 4 illustrates the changes in average entropy for all ABPM samples before and after preprocessing. The original average entropy values were 2.44 bits (SD 0.20 bits) for HR-SBP and 2.48 bits (SD 0.21 bits) for HR-DBP. After KDE smoothing, the average entropy values significantly decreased to 1.55 bits (SD 0.15 bits) and 1.57 bits (SD 0.14 bits), respectively (*p* < 0.001, one-tailed *t*-test). Following further filtering of high-entropy data points, the average entropy values significantly decreased to 0.50 bits (SD 0.25 bits) for HR-SBP and 0.34 bits (SD 0.23 bits) for HR-DBP(*p* < 0.001, one-tailed *t*-test). These results demonstrate that data points generated by simulating from the same BP-HR distribution exhibit improved consistency in their numerical ordering within the overall dataset after entropy reduction preprocessing.

### Transition probabilities of activity and posture exhibit clustering based on statistical metrics

As shown in Fig. 2c,d, the distribution of samples in the P01–P10 scatter plot exhibits self-clustering based on classification results derived from statistical metrics. In these plots, samples labeled 100, 101, 110, and 111 (with sequences containing more than 50% “1”s) cluster in the lower-right region, while samples with fewer than 50% “1”s are positioned in the upper-left region. Furthermore, samples labeled 010, 011, 110, and 111 (which have an average run length of “1”s exceeding two) cluster in the lower region, whereas samples labeled 001, 011, 101, and 111 (whose average run length of “0”s exceeds two) cluster toward the left. Although the distribution patterns for posture states (Fig. 2c) differ from those for activity states (Fig. 2d), samples cluster according to classification results based on statistical metrics. LDA evaluation results show that samples with different statistical-metric-based classifications are well separated by simple straight lines in the P_01_–P_10_ scatter plot, achieving classification accuracies of 90.15% (Activity) and 96.35% (Posture), with corresponding separation ratios of 4.198 (Activity) and 5.357 (Posture), indicating excellent separation and very clear LDA decision boundaries. In the Supplementary Note 5, we provide P01–P10 scatter plots under different statistical metric thresholds along with their classification accuracy and separation ratio, all demonstrating strong clustering effects.

### Entropy reduction preprocessing enhances model generalization and facilitates the prediction of nocturnal hypertension

**Fig. 5:**
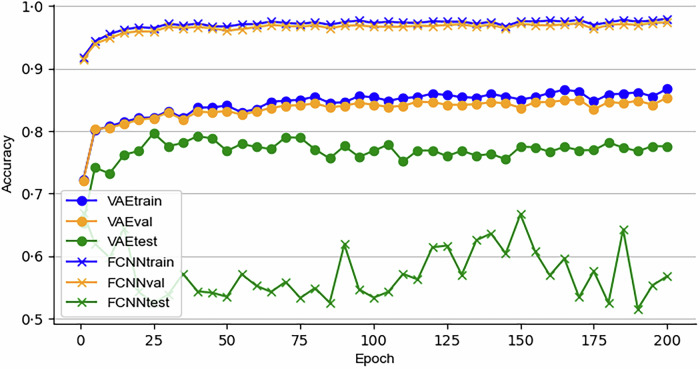
Prediction accuracy of nocturnal hypertension for ABPM-VAE and FCNN models across training, validation, and test sets. **T**he figure displays the performance of the ABPM-VAE and FCNN models on ABPM subsets for predicting nocturnal hypertension. The blue, orange, and green circle lines represent the training, validation, and testing accuracies of the ABPM-VAE model, respectively. Similarly, the blue, orange, and green cross lines represent the corresponding accuracies for the FCNN model. The line plot shows how accuracy changes over the epochs. Since the training and validation subsets originate from the same ABPM recordings, strong performance on these sets alongside poor performance on the test set indicates that the model may have overfitted to individual noise, thereby impairing generalization.

**Fig. 6:**
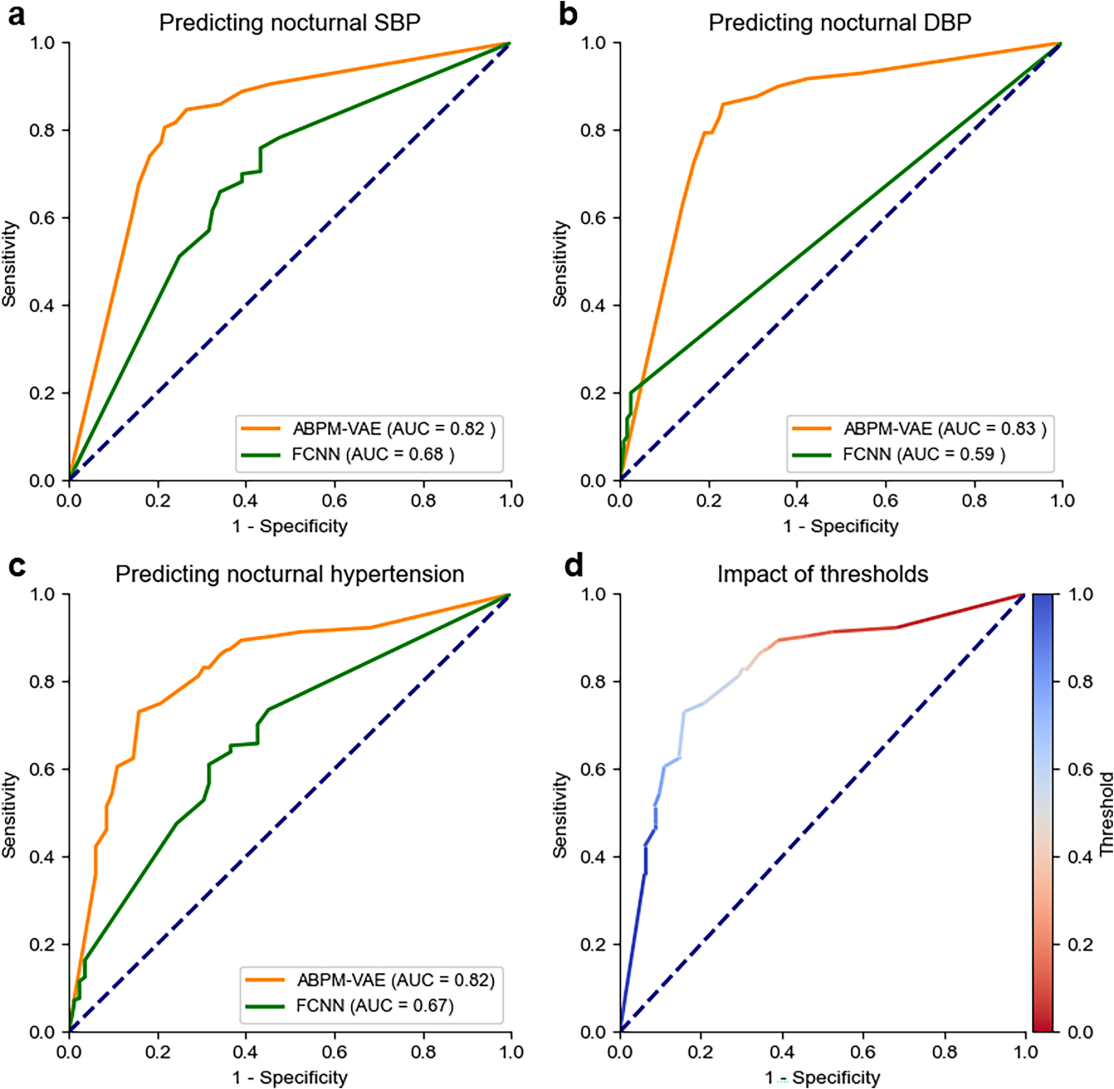
ROC curves of ABPM-VAE and ablation models in test data set. **a** ROC curve for predicting nocturnal systolic hypertension using the ABPM-VAE and ablation screening strategies. Yellow indicates ABPM-VAE; green indicates the FCNN ablation model. **b** ROC curve for predicting nocturnal diastolic hypertension using the ABPM-VAE and ablation screening strategies. Yellow indicates ABPM-VAE; green indicates the FCNN ablation model. **c** ROC curve for predicting overall nocturnal hypertension using the ABPM-VAE and ablation screening strategies. Yellow indicates ABPM-VAE; green indicates the FCNN ablation model. **d** ROC curve for the ABPM-VAE screening strategy showing the impact of thresholds on sensitivity and specificity. The color gradient represents thresholds, with red for lower values and blue for higher values. When the ROC threshold was set to 0.6, the ABPM-VAE model achieved a PPV of 92.12%, a NPV of 55.20%, a sensitivity of 0.73, and a specificity of 0.84. *n* = 2874 biologically independent patient samples.

The prediction accuracy of ABPM subsets is shown in Fig. 5. After 100 epochs, the training set results stabilized, and the FCNN achieved perfect accuracy in both the training set (0.98; 95% CI, 0.97–0.98) and validation set (0.97 95% CI, 0.96–0.97), but the ABPM-VAE model reached approximately 87% accuracy in the training set (0.86; 95% CI, 0.85–0.87) and validation set (0.84; 95% CI, 0.84–0.85). However, on the test set, the FCNN’s accuracy dropped to 58% (0.58; 95% CI, 0.52–0.66), whereas the ABPM-VAE maintained 77% accuracy (0.77; 95% CI, 0.75–0.78). This demonstrates that the ABPM-VAE model exhibited significantly better generalization capability (*p* < 0.001, one-tailed *t*-test). More detailed MSE results comparing ABPM-VAE and FCNN performance are provided in Supplementary Fig. 4. Furthermore, prediction results for nocturnal SBP/DBP from the resultstest set reinforce these findings. After 200 epochs, the ABPM-VAE model demon- strated strong discriminative ability for nocturnal systolic hypertension (AUC 0.82; 95% CI, 0.77–0.87) (Fig. 6a) and nocturnal diastolic hypertension (AUC 0.83; 95% CI, 0.79–0.89) (Fig. 6b), whereas the FCNN performed poorly (AUC 0.68 and 0.59, respectively). Moreover, the ABPM-VAE model exhibited robust performance for predicting nocturnal hypertension (AUC 0.82; 95% CI, 0.77–0.88) (Fig. 6c), compared to an AUC of 0.67 (95% CI, 0.61–0.74) for the FCNN. When the ROC threshold was set to 0.6, the ABPM-VAE model achieved a PPV of 92.12%, a NPV of 55.20%, a sensitivity of 0.73, and a specificity of 0.84 (Fig. 6d).

We then compared the consistency of nocturnal hypertension predictions between the FCNN and ABPM-VAE models under different within-hour measurement selection strategies, namely, “first" selecting the first record within each hour, “last" selecting the last record, “alt_first" alternating between first for odd-numbered selections and last for even-numbered selections, and “alt_last" alternating between last for odd-numbered selections and first for even-numbered selections, using pairwise Cohen’s Kappa analysis. For both models, classification agreement rates across these strategies exceeded 82%, with the lowest Cohen’s Kappa coefficient being 0.636, indicating substantial agreement, and all pairwise comparisons reaching substantial or almost perfect agreement levels. These results demonstrate that predictions from both the FCNN and ABPM-VAE models are robust to variations in within-hour selection of daytime readings. The specific agreement rates and Cohen’s Kappa values for the ABPM-VAE and FCNN models in SBP/DBP across the various within-hour measurement selection strategies are provided in the Supplementary Note 7.

**Fig. 7:**
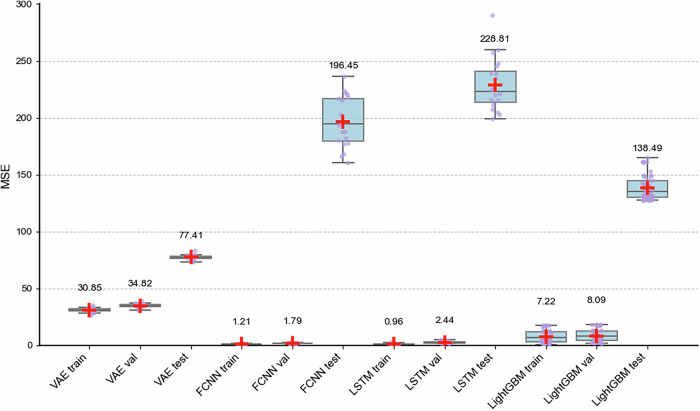
MSE performance of ABPM-VAE, ablation, and baseline models across training, validation, and test data sets. **A**ll boxes are rendered in a uniform light blue hue. The centre line of each box denotes the median, while a red plus sign marks the mean; the mean value is annotated immediately above each box. The upper and lower edges of each box correspond to the first and third quartiles, respectively, and the whiskers extend to ±1.5 times the interquartile range. Light purple dots represent the performance of each method under different test epochs or parameter settings. The ABPM-VAE model achieved the lowest MSE of 77.41 (SD 1.97), (*n* = 20 epoches), significantly outperforming all other models (*p* < 0.001, two-tailed *t*-test). In comparison, the LightGBM model yielded an MSE of 138.49 (SD 10.86), (*n* = 48 parameter settings), the FCNN ablation model 196.45 (SD 22.15), (*n* = 20 epoches), and the LSTM model 228.81 (SD 22.92), (*n* = 20 epoches).

Finally, comparing the baseline models LightGBM and LSTM against the ABPM-VAE model and FCNN using MSE on the test set showed that the ABPM-VAE model achieved the lowest MSE of 77.41 (1.97), significantly outperforming all other models (*p* < 0.001, two-tailed *t*-test). In comparison, the LightGBM model yielded an MSE of 138.49 (10.86), the FCNN ablation model 196.45 (22.15), and the LSTM model 228.81 (22.92) (Fig. 7). These results further confirm the superior predictive performance and generalization capability of the ABPM-VAE model. Detailed MAE, RMSE, and MSE performance for nocturnal hypertension prediction using the LSTM and LightGBM models across the training, validation, and test sets is provided in Supplementary Notes 6 and 7, respectively.

### Superior classification performance of nocturnal hypertension over dipping patterns

Nocturnal hypertension and dipping patterns were derived from the same predicted nocturnal BP values generated by ABPM-VAE, but differ in their classification approach: fixed thresholds for nocturnal hypertension (mean SBP ≥120 mmHg or DBP ≥70 mmHg) versus a relative threshold for non-dipping (<10% nocturnal SBP fall relative to individual daytime mean). In the test set, ABPM-VAE achieved an accuracy of 0.77 and AUC of 0.82 for nocturnal hypertension, compared with 0.70 and AUC of 0.63 for dipping patterns (Supplementary Note 8). Noise-sensitivity simulations further confirmed the greater vulnerability of abnormal dipping pattern assessment: across different noise levels (*σ* = 1–20 mmHg), both the accuracy and AUC of dipping patterns were significantly lower than those of nocturnal hypertension, with mean differences of −0.023 (standard error 0.001) for accuracy and -0.044 (standard error 0.008) for AUC (two-tailed *t*-test, both *p* < 0.001) (Supplementary Fig. 7 and Table S6). The AUC difference was significantly greater than the accuracy difference (two-tailed paired *t*-test, *p* < 0.001) (Supplementary Fig. 8 and Table S7). Therefore, ABPM-VAE provides reliable classification of nocturnal hypertension, while dipping pattern assessment, due to its heightened sensitivity to prediction uncertainty, requires more cautious interpretation.

### Reliable correction and predictive performance of BP variability based on ABPM-VAE density maps

The ABPM-VAE model outputs joint BP-HR probability density maps. To estimate BP variability, the quantitative relationship between CV(map) derived from the probability density map and CV(sample) derived from raw measurements was first established. We mathematically derived a proportional scaling relationship between CV(map) and CV(sample), and the theoretical derivation identified a proportional scaling relationship between the map-derived and sample-derived coefficients of variation: CV(sample) = *k* × CV(map). The scaling coefficient *k* is governed by the kernel bandwidth *h*, which was adaptively selected according to sample size. For daytime periods (*n* = 16 samples), *h* = 0.630 yielded a theoretical *k* of 0.859; for nighttime periods (*n* = 8 samples), *h* = 0.707 yielded *k* = 0.832, with the detailed derivation provided in the Supplementary Note 9.

Empirical validation via Bland–Altman analysis across 12 subsets (training/validation/test × daytime/nighttime × SBP/DBP) confirmed excellent agreement using the theoretical coefficients, with mean bias <−0.45% and 95% limits of agreement (LoA) width <1.55% across all subsets. Joint least-squares fitting further optimized empirical scaling coefficients *k*: daytime SBP *k* = 0.890, DBP *k* = 0.894; nighttime SBP *k* = 0.842, DBP *k* = 0.843. Application of these empirical *k* values improved agreement (mean bias ≈0%, 95% LoA width <1.30%), enabling virtually unbiased estimation of true BP variability from the maps, with the detailed derivation provided in the Supplementary Note 10. Thus, CV(sample) was computed as *k* × CV(map).

Performance of the ABPM-VAE model in predicting nocturnal BP variability was evaluated by comparing the corrected CV derived from predicted CV(map) against the true CV(sample). Mean absolute errors were 6.28% (SD 3.43%) for SBP CV and 6.37% (SD 4.29%) for DBP CV, indicating systematic overestimation (Supplementary Table S10). Therefore, while a clear mathematical relationship exists between the true CV(map) and true CV(sample), a reliable correspondence between the predicted CV(map) and true CV(sample) has not yet been established.

### Vanilla gradient saliency reveals contour-dominant attention in ABPM-VAE for nighttime prediction

**Fig. 8:**
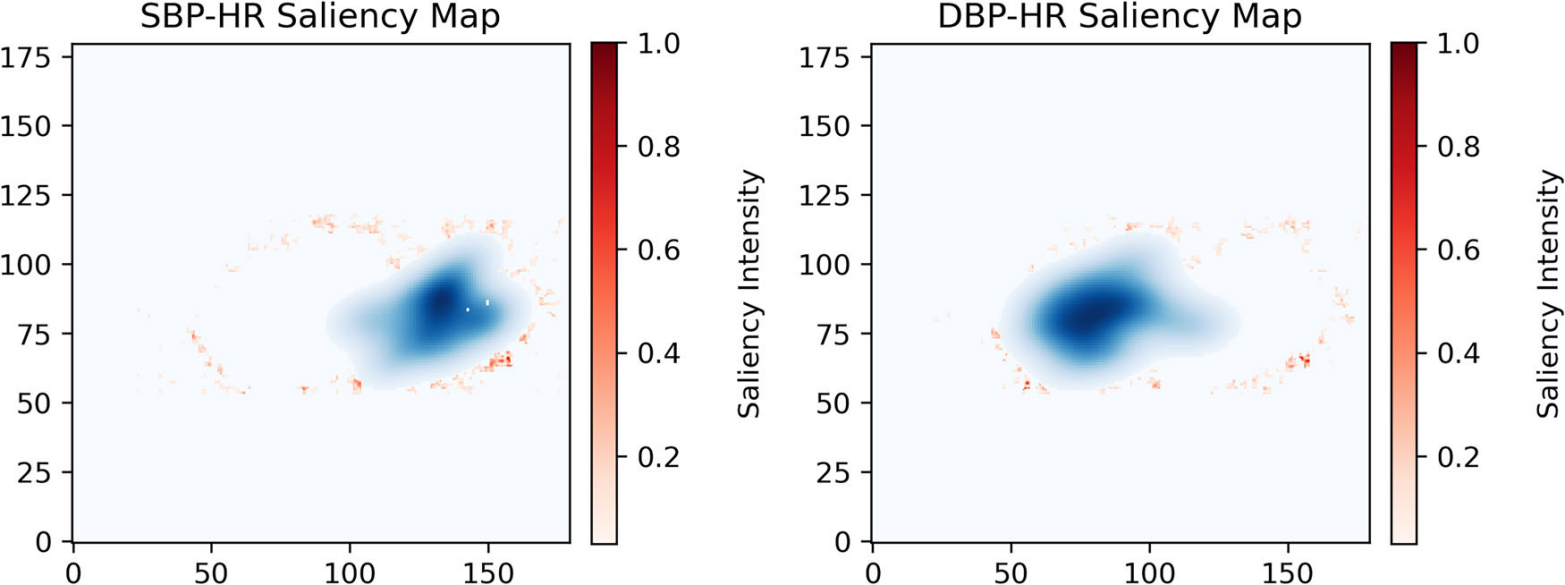
Vanilla gradient saliency maps of ABPM-VAE for nighttime BP-HR density maps prediction. Vanilla saliency maps are computed as the absolute input gradients with respect to predicted nighttime BP-HR distributions, normalized for visualization. High-saliency regions (red) form a consistent band aligning closely with the contours and outer boundaries of daytime SBP-HR (left) and DBP-HR (right) joint probability density maps.

Vanilla saliency maps revealed that the ABPM-VAE model focuses on the contours and overall morphology of the daytime SBP-HR and DBP-HR joint probability density distributions. As illustrated in Fig. 8, saliency maps were generated by computing the absolute values of the input gradients with respect to the predicted nighttime distributions, followed by normalization for visualization. High-saliency regions (highlighted in red) form a band that surrounds and closely aligns with the outer boundaries of both the daytime SBP-HR and DBP-HR density maps. These findings indicate that the model primarily attends to the shape, spread, and boundary characteristics of the daytime joint probability density distributions when inferring the corresponding nighttime BP-HR distributions.

### Targeted perturbation reveals activity/posture dependent modulation of nocturnal BP in ABPM-VAE

Targeted perturbation experiments were conducted to quantify the influence of activity and posture transition probabilities on predicted nocturnal BP. By forcing the input activity/posture transition probabilities (P01 and P10) to fixed values in the test set, four scenarios were designed. The mean differences in predicted nocturnal SBP/DBP (perturbed minus original prediction; mean (SEM)) are as follows, with statistical significance assessed by two-tailed *t*-tests:

Scenario 1: Daytime forced to resting/supine state (P01=0, P10=1; indicating no transition from resting/supine to active/standing and full transition from active/standing to resting/supine); nighttime unchanged. The perturbed predictions are higher than the original predictions: SBP 2.268 (0.045) mmHg (*p* < 0.001); DBP 1.543 (0.033) mmHg (*p* < 0.001).

Scenario 2: Nighttime forced to resting/supine state (P01 = 0, P10 = 1); daytime unchanged. The perturbed predictions are lower than the original predictions: SBP −0.268 (0.021) mmHg (*p* < 0.001); DBP −0.070 (0.015) mmHg (*p* < 0.001).

Scenario 3: Daytime forced to active/standing state (P01 = 1, P10 = 0; indicating full transition from resting/supine to active/standing and no transition from active/standing to resting/supine); nighttime unchanged. The perturbed predictions are lower than the original predictions: SBP −4.042 (0.069) mmHg (*p* < 0.001); DBP −2.653 (0.055) mmHg (*p* < 0.001).

Scenario 4: Nighttime forced to active/standing state (P01 = 1, P10 = 0); daytime unchanged. The perturbed predictions are higher than the original predictions: SBP 8.067 (0.109) mmHg (*p* < 0.001); DBP 5.897 (0.087) mmHg (*p* < 0.001).

This indicates that the ABPM-VAE model has learned that daytime active/standing is associated with lowered nocturnal BP and nighttime active/standing is associated with elevated nocturnal BP, and vice versa. Additionally, the magnitude of change is consistently greater for SBP than for DBP (*p* < 0.001).

## Discussion

ABPM holds an irreplaceable role in the diagnosis and management of hypertension, particularly nocturnal hypertension. However, cuff inflation noise and arm compression discomfort render nighttime ABPM intolerable for 8.8% of the general population, with even higher rates among the nearly 60 million patients with dementia^3,4,48^. Given that approximately 1.28 billion adults aged 30–79 years worldwide have hypertension, the number of hypertensive patients unable to tolerate ABPM reaches hundreds of millions^49^. As noted in the introduction, previous studies using home BP measurements, demographic characteristics, medical history, and medication data have shown suboptimal performance in predicting nocturnal hypertension. To address this gap, we developed a deep learning model for nocturnal BP prediction based on a preprocessing framework that integrates KDE with entropy reduction techniques. Importantly, this method requires no additional data collection and clinicians can leverage existing recorded ABPM data to identify high-risk individuals, thereby improving detection rates in this group and meeting a clear unmet clinical need.

The study revealed several key findings. First, KDE-based entropy reduction preprocessing effectively alleviates time-series instability arising from short-term fluctuations and aliasing in ABPM data, yielding substantial gains in predictive performance and clinical screening utility. Second, by prioritizing boundary and contour features of probability density distributions over local peaks, the model captures global structural information, enhancing both accuracy and interpretability. Finally, targeted perturbation experiments demonstrated that transition probabilities of activity and posture, when used as embedding vectors, exert influences on predicted nocturnal BP that are consistent with well-established clinical evidence, thereby supporting the conclusion that ABPM-VAE has captured physiological patterns consistent with established clinical evidence.

Significant short-time-scale fluctuations are aliased into the BP-HR measurements in ABPM data. As a result, even within the same period for the same patient, different sampling outcomes occur, leading to instability in the numerical ordering of data points and compromising the stability of time series features. To address this, we propose an entropy reduction preprocessing method that smooths BP-HR using Gaussian KDE to generate density maps and clip high entropy regions. Previous studies have demonstrated that features extracted from surface electromyography signals preprocessed with KDE can be used to classify hand motion using an XGBoost decision tree model^50^. Similarly, KDE-preprocessed electroencephalography features have been applied to emotional state classification with artificial neural networks^51^. Recently, circadian rhythm features of vital signs processed via KDE have been used to assess physiological stability and provide early warnings for severe adverse events^52^. However, previous studies focused on analyzing statistical features derived from KDE probability density estimates of 1D time series, rather than utilizing the probability density distributions for prediction.

In this study, we extended KDE from one dimension to two-dimensional joint BP-HR probability density maps. Entropy-based preprocessing markedly reduces distributional uncertainty (SBP-HR entropy from 2.44 bits to 0.50 bits; DBP-HR from 2.48 bits to 0.34 bits), narrowing data-point dispersion from approximately 5/10 bins to fewer than 2/10 bins. The resulting cropped density maps, combined with activity and posture transition probabilities, serve as inputs to ABPM-VAE, enabling generation of nocturnal probability density profiles. This approach achieves superior predictive performance compared with conventional methods (AUC 0.82 vs. 0.69), detects 73% of patients with nocturnal hypertension without additional burden on patients or clinicians (PPV 92.12%, NPV 55.20%), and is thus suitable for effective screening but not for exclusionary screening.

We validated the effectiveness of entropy reduction preprocessing through ablation experiments. The ABPM-VAE model features two paths: the upper path compresses and reconstructs probability density maps via convolution and deconvolution without learning nocturnal hypertension features, while the lower path uses backpropagation solely through the FCNN to extract hypertension-specific features. This design decouples compression from feature learning, so both ABPM-VAE and the ablation model depend only on the FCNN for nocturnal hypertension prediction. Only ABPM-VAE receives entropy-reduced inputs, enabling isolated evaluation of the preprocessing effect. Results show ABPM-VAE outperforms the FCNN model in predicting nocturnal systolic/diastolic hypertension and overall nocturnal hypertension, confirming the preprocessing improves predictive performance. Performance across training, validation, and test sets reveals severe overfitting with original data, which entropy reduction mitigates. The ablation model excelled on the validation set (same patients as training), indicating memorization of individual traits, but performed poorly on the test set (different patients), showing limited generalization. Sensitivity analysis of within-hour ABPM measurement selection strategies confirmed the model’s robustness to daytime sampling intervals and occasional missing readings, reinforcing its clinical practicality.

Unlike traditional approaches relying on point estimates or shallow statistical features, this method preserves global distributional structure to improve model robustness and interpretability. Furthermore, embedding transition probabilities across activity and posture states revealed regulatory patterns highly consistent with established clinical physiological mechanisms. In Vanilla Gradient Saliency analysis, we observed that the ABPM-VAE model primarily focuses on the boundary features of the daytime joint probability density distribution when inferring the nighttime BP-HR distribution. This strong emphasis on contours and edge regions, rather than on central high-density areas or isolated point estimates, indicates that ABPM-VAE captures the global structural information of the BP-HR probability distribution, instead of relying on local density peaks. Previous studies have shown that this ability to learn global structures can improve both the performance and clinical interpretability of deep learning models^53–55^. Our entropy calculations on the numerical ordering of measurement points further confirm that entropy-reduction preprocessing mitigates the fluctuation of data points. The Vanilla Gradient Saliency results additionally demonstrate that ABPM-VAE benefits from this reduced variability by focusing on and learning global structural information rather than isolated individual data points.

On the other hand, activity and posture are represented as sparse binary time series that are susceptible to the vanishing gradient problem during model training. A common solution for handling such discrete signals is to embed them into low-dimensional continuous vectors, which can capture latent structures and improve model performance^21–23^. However, activity and posture time series lack clear clinical indicators, making it challenging to map these signals to embedding vectors based on clinical relevance. To address this, we extract the transition probabilities P01 and P10 from activity and posture time series as embedding vectors, preserving the statistical features of the sequence. This strategy converts the binary signal sequence into a statistically meaningful low-dimensional continuous embedding vector. As shown in the P01–P10 scatter plot, samples with similar statistical characteristics cluster together, facilitating better segmentation and pattern recognition by the model.

Targeted perturbation experiments further reveal the influence of activity and posture transition probabilities P01 and P10 on predicted nocturnal BP: daytime active/standing is associated with lowered predicted nocturnal BP, whereas nighttime activity is associated with elevated predicted nocturnal BP, and vice versa. Additionally, the magnitude of these changes is consistently greater for SBP than for DBP. These patterns align closely with established clinical evidence. Regular aerobic exercise is recommended to reduce cardiovascular risk, in part by lowering resting BP. A single bout of moderate-intensity acute aerobic exercise can induce post-exercise hypotension, resetting baroreflexes to a lower BP level, with reductions typically in the range of 2–4 mmHg in SBP and smaller changes in DBP, as documented in multiple guidelines^4,56,57^. This matches our ABPM-VAE Targeted Perturbation findings that daytime active/upright lowers predicted nocturnal BP, predominantly SBP. Conversely, BP rises acutely during dynamic and static exercise, more markedly in SBP than DBP, consistent with our observation that nighttime active/upright elevates predicted nocturnal BP^57^. This concordance with physiology and clinical guidelines—where regular daytime activity promotes lower resting BP while nocturnal activity or poor sleep habits increases risk—supports that the model has captured physiological patterns consistent with established clinical evidence.

Despite the promising performance of the ABPM-VAE model in predicting nocturnal hypertension and its successful establishment of a mathematical proportional relationship between the predicted probability density maps and true BP variability, this study has several limitations. First, the classification of dipping patterns is highly sensitive to prediction errors, representing a clinically relevant constraint. Although both nocturnal hypertension and abnormal dipping patterns are derived from the same predicted nocturnal SBP/DBP values, dipping pattern classification relies on an individual relative threshold (<10% nocturnal decline), resulting in markedly inferior performance compared with the fixed-threshold classification of nocturnal hypertension. Noise sensitivity simulations further confirmed that, at equivalent levels of prediction error, dipping pattern accuracy and AUC were substantially lower than those for nocturnal hypertension. This observation aligns with the poor real-world concordance reported for cuffless devices (such as the Aktiia bracelet) in assessing dipping patterns, indicating that dipping pattern remains an unreliable clinical phenotype under current prediction accuracy^58,59^. Clinicians should therefore exercise caution when interpreting model-derived dipping classifications. It is also worth noting that although Aktiia has been validated under the AAMI/ESH/ISO Universal Standard (ISO 81060-2:2018) and performs reliably during daytime measurements, its effectiveness for nocturnal BP monitoring remains questionable. Clinical reports utilizing ABPM as the reference standard for mean nocturnal BP have shown that Aktiia yields mean errors of 13.65 mmHg, 8.3 mmHg, and −4.5 mmHg^58–61^. In comparison, the ABPM-VAE model achieved a mean error of -0.61 (SD 8.57) mmHg. Although the SD (8.57 mmHg) slightly exceeded the 8 mmHg threshold, the model demonstrates significant clinical utility as a predictive screening tool, particularly given the inherent challenges of cuffless nocturnal blood pressure monitoring. Second, predictions of BP variability exhibit systematic bias. Although we theoretically derived and validated via Bland-Altman analysis on the real dataset—a fixed proportional scaling relationship between the CV of the filtered density map CV(map) and that of the raw samples CV(sample), direct computation of CV from the model-generated predictive density maps reveals a pronounced positive systematic bias. This overestimation primarily stems from an inherent property of standard VAEs under generative mode, where MSE-driven training encourages mean regression toward all plausible target values when the model is uncertain about fine details of the density map. Consequently, the generated distributions become blurred, with minimal impact on mean estimation but substantial loss of fidelity in higher-order moments such as variance or variability. This limitation is a common challenge for current VAE architectures in medical generation tasks that require precise capture of distribution tails and higher-order statistical features. In summary, although the ABPM-VAE model advances nocturnal mean BP prediction and establishes a mathematical linkage to variability, the intrinsic instability of dipping pattern classification and the systematic overestimation of variability in the VAE generative mode limit its direct application to comprehensive clinical phenotyping.

Overall, we developed an ABPM-VAE model incorporating entropy reduction preprocessing to predict nocturnal hypertension, achieving superior performance compared with prior methods based on demographic features. By capturing the global structural information of daytime BP–HR joint probability density maps and integrating the activity/posture transition probabilities, which align with established physiology and clinical guidelines, the model generates nocturnal probability density maps, markedly enhancing its interpretability and clinical credibility. Our study aligns well with real-world clinical needs: it requires no additional data collection, imposes no extra burden on patients or clinicians, and enables high-risk alerts for individuals intolerant to nocturnal ABPM, thereby assisting physicians in deciding whether further monitoring is warranted for screened high-risk patients and serving as a valuable complement to ABPM monitoring.

## Supplementary information

Transparent Peer Review file

Supplementary Information

Description of Additional Supplementary files

Supplementary Data 1

Supplementary Data 2

Supplementary Data 3

Supplementary Data 4

Supplementary Data 5

Supplementary Data 6

Supplementary Data 7

Supplementary Data 8

Supplementary Data 9

Supplementary Data 10

## Data Availability

The numerical source data supporting the primary charts and statistical analyses are provided as Supplementary Data in CSV format. The anonymized patient demographic information and ABPM data used in this study are publicly available on Figshare at 10.6084/m9.figshare.29431085. This data is available for academic and non-commercial research purposes^62^.

## Code availability

The code for implementing the ABPM-VAE model and related preprocessing algorithms is publicly available at 10.6084/m9.figshare.29431085^62^. This code repository will include documentation and annotations to ensure reproducibility. Access details will be provided after publication. The code is intended for academic and non-commercial use.
