# Supplementary material for: Deep learning prediction of nocturnal hypertension for patients intolerant to ambulatory blood pressure monitoring
Source: Commun Med (Lond). 2026 May 8;6:396. doi: 10.1038/s43856-026-01639-x (PMC13369173; doi:10.1038/s43856-026-01639-x)
Supplement: Supplementary file 2 — Supplementary Information [file 43856_2026_1639_MOESM2_ESM.pdf]

Supplementary Notes for: Deep Learning Prediction of Nocturnal Hypertension for Patients Intolerant to ABPM

Contents

Supplementary Note 1: Patient Baseline Demographics and ABPM Profiles 2

Supplementary Note 2: Entropy Analysis of DBP-HR Distributions Before and After Preprocessing 4

Supplementary Note 3: Estimation of Short-Term Fluctuations in SBP, DBP, and HR 6

Supplementary Note 4: FCNN Architecture for Nocturnal Hypertension Prediction 7

Supplementary Note 5: Scatter Plots of $P_{01} - P_{10}$ Under Different Statistical Metric Thresholds and LDA Evaluation Results 8

Supplementary Note 6: MSE performance of FCNN, LSTM, and LightGBM 10

Supplementary Note 7: RMSE and MAE Performance of ABPM-VAE, Ablation, and Baseline Models across Training, Validation, and Test Data Sets 13

Supplementary Note 8: Classification Agreement Across Different Daytime Reading Selection Strategies 15

Supplementary Note 9: Classifications of Dipping-Pattern vs Nocturnal Hypertension 16

Supplementary Note 10: Analytical Estimation and Joint Least-Squares Fitting of Variance Increase After Filtering 20

Supplementary Note 11: Empirical Validation via Bland–Altman Analysis Across 12 Subsets 22

Supplementary Note 1: Patient Baseline Demographics and ABPM Profiles

Table S1. Baseline Characteristics of the Study Population

Category	Characteristic	Nocturnal SBP (n=1592)	Nocturnal DBP (n=1703)	Total (n=2874)
Demographics				
	Age, mean (SD), y	55.7 (15.9)	51.3 (14.5)	54.2 (15.7)
	Female, n	756	716	1382
	Male, n	836	987	1492
Medical history				
	Coronary Heart Disease	428	330	708
	Heart Valvular Disease	344	281	569
	Heart Failure	114	71	162
	Atrial Fibrillation	58	50	108
	Diabetes Mellitus	283	234	411
	Renal Insufficiency	134	93	187
	Thyroid Dysfunction and Nodule	197	194	362
	Hyperuricemia	286	291	504
	Sleep Apnea Syndrome	30	35	44
	Sleep Disorders and Anxiety	136	133	275
	Renal Artery Stenosis	114	73	165
Medication history				
	ACEI	83	66	128
	ARBs	207	187	382
	CCB	297	268	513
	Diuretics	87	78	180
	Beta Blockers	168	129	308
Laboratory results, mean (SD)				
	eGFR (ml/min/1.73m²)	94.0 (27.4)	100.6 (26.0)	96.6 (26.0)
	LDL (mmol/L)	2.6 (0.8)	2.7 (0.8)	2.7 (0.9)
	Sodium (mmol/L)	144.1 (3.2)	143.8 (3.2)	143.8 (3.2)
	Potassium (mmol/L)	4.0 (1.9)	4.1 (2.9)	4.1 (2.2)
	BNP (pg/ml)	125.3 (274.4)	107.0 (276.0)	115.2 (310.6)
	TSH (mIU/L)	2.3 (1.1)	2.2 (1.1)	2.2 (1.0)
	Free Thyroxine (pmol/L)	16.2 (2.4)	16.3 (2.4)	16.3 (2.4)
	Uric Acid (μmol/L)	369.3 (108.8)	375.5 (106.4)	368.7 (105.3)
Echocardiography, mean (SD)				

(Continued on next page)

(Continued from previous page)

Category	Characteristic	Nocturnal SBP (n=1592)	Nocturnal DBP (n=1703)	Total (n=2874)
	Left atrium diameter (mm)	38.1 (5.5)	37.7 (5.6)	37.6 (5.4)
	Left ventricle diameter (mm)	47.3 (5.1)	47.4 (5.1)	47.0 (5.0)
	Right atrium diameter (mm)	46.9 (5.8)	46.9 (5.7)	46.7 (5.6)
	Right ventricle diameter (mm)	20.5 (2.8)	20.5 (2.8)	20.4 (2.7)
	Ejection fraction (%)	64.3 (6.0)	64.4 (5.6)	64.5 (6.0)
	Ascending aorta diameter (mm)	34.9 (4.1)	34.7 (4.2)	34.4 (4.2)

The table summarizes demographic, clinical, laboratory, and echocardiographic characteristics for patients included in the nocturnal SBP and DBP analyses, as well as the overall population. Values are presented as mean (standard deviation) for continuous variables and counts for categorical variables. Abbreviations: ACEI, angiotensin-converting enzyme inhibitor; ARBs, angiotensin II receptor blockers; BNP, B-type natriuretic peptide; CCB, calcium channel blockers; eGFR, estimated glomerular filtration rate; LDL, low-density lipoprotein; TSH, thyroid-stimulating hormone.

Table S2. Descriptive Statistics of Patient ABPM Data

Measure	Day	Night	Overall	Abs Diff (D–N)
SBP (mmHg)	130.49 (12.98)	123.50 (15.20)	129.08 (12.86)	9.81 (7.11)
DBP (mmHg)	80.53 (10.43)	72.89 (10.72)	78.99 (10.14)	8.52 (5.64)
HR (bpm)	76.00 (10.13)	64.82 (9.48)	73.75 (9.67)	11.40 (5.98)
Supine (%)	54.07 (18.49)	87.24 (18.51)	60.76 (15.78)	36.05 (19.42)
Resting (%)	80.54 (13.73)	96.50 (8.12)	83.77 (11.61)	16.80 (12.25)

The table presents the descriptive statistics of patient ABPM data, with values reported as mean (standard deviation). Day (06:00–22:00) and Night (22:00–06:00) represent the respective recording periods. *Abs Diff (D–N)* denotes the absolute difference between day and night means. *Supine (%)* and *Resting (%)* indicate the percentage of measurements taken in the supine position and during a no-activity state, respectively.

Supplementary Note 2: Entropy Analysis of DBP-HR Distributions Before and After Preprocessing

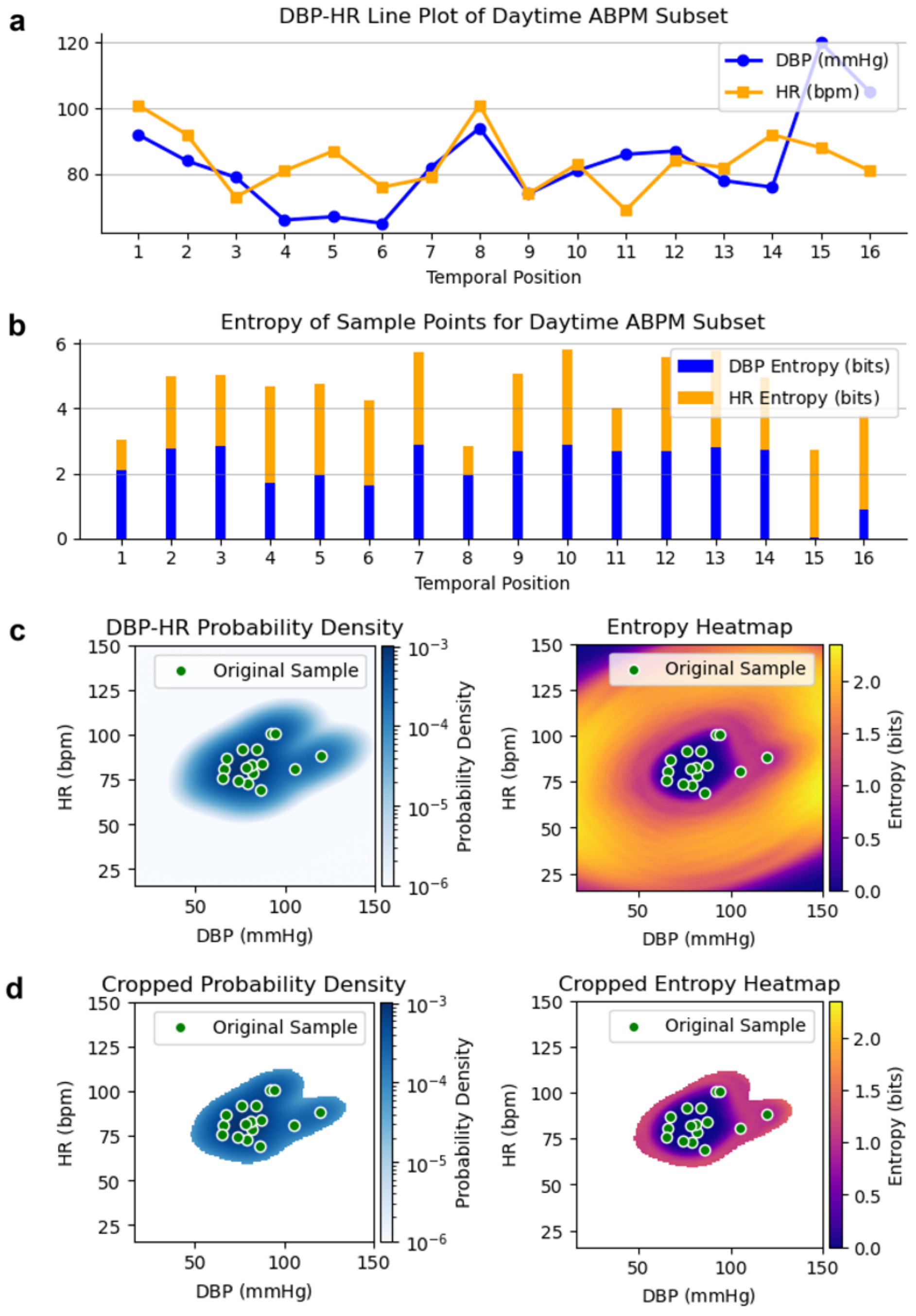

Supplementary Figure 1. Entropy of DBP-HR Data Point Bin Assignments Before and After Entropy Reduction Preprocessing

a, Line plot for the Daytime ABPM Subset (ID: 1620#2021-03-20). The blue line represents the DBP recorded between 6:00 AM and 10:00 PM, and the yellow line depicts the HR recorded over the same time period. **b**, Bar plot showing the entropy of sample points for DBP-HR in the Daytime

ABPM Subset. The blue bars represent the entropy of DBP, while the yellow bars represent the entropy of HR. **c**, The left panel shows the probability density distribution generated after applying KDE estimation to the DBP-HR data, where darker colors indicate higher probability densities. The right panel presents the corresponding Entropy Heatmap generated using Monte Carlo simulation and the 10-bin method, where darker colors indicate lower entropy values. Green points represent the original DBP-HR data. **d**, The left panel displays the probability density distribution obtained by cropping grid points with probability densities below 5% of the maximum value from the distribution in panel c. The right panel presents the corresponding Entropy Heatmap. Green points represent the original DBP-HR data.

Supplementary Note 3: Estimation of Short-Term Fluctuations in SBP, DBP, and HR

ABPM recordings of BP and HR exhibit short-time-scale fluctuations that lead to aliasing, superimposed on more stable long-time-scale trends. We simulated this process using a Monte Carlo approach, assuming that each measurement follows a normal distribution $N(\mu, \sigma^2)$, where μ is the ABPM-recorded value and σ is the empirical standard deviation. Here, we show the estimation process for the parameter σ of SBP, DBP, and HR.

Consider a time series consisting of three consecutive observations, denoted A, B, and C. We assume that the observations are independent and normally distributed as:

$$A \sim N(\mu_1, \sigma^2), \quad B \sim N(\mu_2, \sigma^2), \quad C \sim N(\mu_3, \sigma^2),$$

where μ_1, μ_2, and μ_3 represent the expected mean values at the respective measurement points, and σ denotes the standard deviation of short-time-scale fluctuations. For simplicity, we assume that σ is constant across patients and timepoints.

Given the absence of monotonic temporal trends in population-level BP-HR recordings, we assume that the expected differences between adjacent means are centered at zero:

$$\mu_1 - \mu_2 \sim N(0, \sigma_l^2), \quad \mu_2 - \mu_3 \sim N(0, \sigma_l^2),$$

where σ_l represents long-time-scale physiological variation, also assumed to be constant.

Under this model, the Lag-1 and Lag-2 differences follow:

$$A - B \sim N(0, 2\sigma^2 + \sigma_l^2), \quad A - C \sim N(0, 2\sigma^2 + 2\sigma_l^2).$$

Thus, by computing the empirical variances of the Lag-1 difference ($A - B$) and the Lag-2 difference ($A - C$), we can estimate σ_l^2 as their difference. Solving the resulting equations allows us to recover σ, the standard deviation associated with short-time-scale fluctuations.

Although simplified, this model yields estimates consistent with prior experimental data. Specifically, the estimated short-term standard deviations for SBP, DBP, and HR were 8.17 mmHg, 6.69 mmHg, and 5.67 bpm, respectively—close to previously reported experimental values of 8.09 mmHg, 5.65 mmHg, and 4.40 bpm. Importantly, the conclusions of the Monte Carlo simulation for entropy analysis remain unchanged regardless of whether estimated or experimental parameter sets are used.

Table S3. Variance Decomposition Based on Lag-1 and Lag-2 Differences for SBP, DBP, and HR

**Signal**	**Difference**	**Var**	σ_l^2	σ^2	σ
SBP	Lag-1 diff	184.62	50.96	66.83	8.17
	Lag-2 diff	235.58			
DBP	Lag-1 diff	121.12	31.67	44.72	6.69
	Lag-2 diff	152.79			
HR	Lag-1 diff	91.73	27.33	32.20	5.67
	Lag-2 diff	119.06			

The table presents the empirical variances of Lag-1 and Lag-2 differences for SBP, DBP, and HR. The long-time-scale variance component σ_l^2 is estimated as the difference between the Lag-1 and Lag-2 variances. The short-term variance σ^2 is obtained by solving the system of equations derived from the hierarchical model. The corresponding standard deviation σ quantifies the magnitude of short-time-scale physiological fluctuations.

Supplementary Note 4: FCNN Architecture for Nocturnal Hypertension Prediction

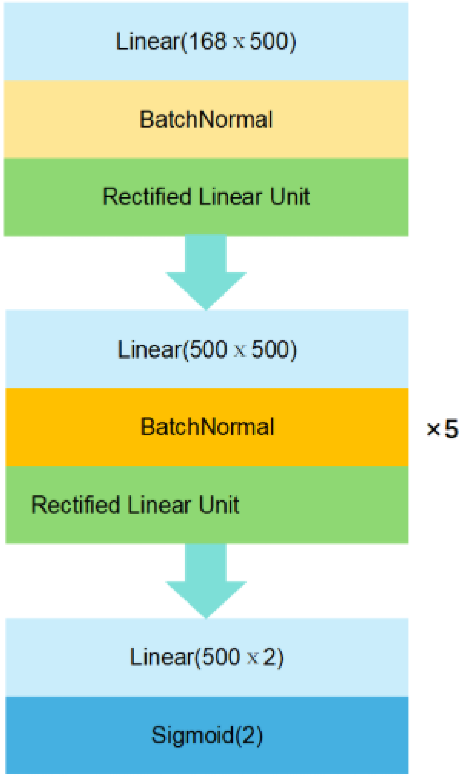

Supplementary Figure 2. FCNN architecture

The FCNN model takes as input the timestamp, SBP, DBP, HR, activity state, positional state, and a binary missingness indicator (1 for daytime measurements and 0 for withheld nighttime values). The output labels are the average nocturnal SBP and DBP, and nocturnal hypertension labels were then derived from FCNN outputs using the same clinical thresholds as the ABPM-VAE model (SBP 120 mmHg or DBP 70 mmHg for positive labels). The model is trained using MSE as the loss function between outputs and true nocturnal SBP/DBP labels. All parameters were optimized via backpropagation. The network is optimized using the Adam optimizer with a learning rate of 0.0001, and MSE is used as the loss function.

Supplementary Note 5: Scatter Plots of $P_{01} - P_{10}$ Under Different Statistical Metric Thresholds and LDA Evaluation Results

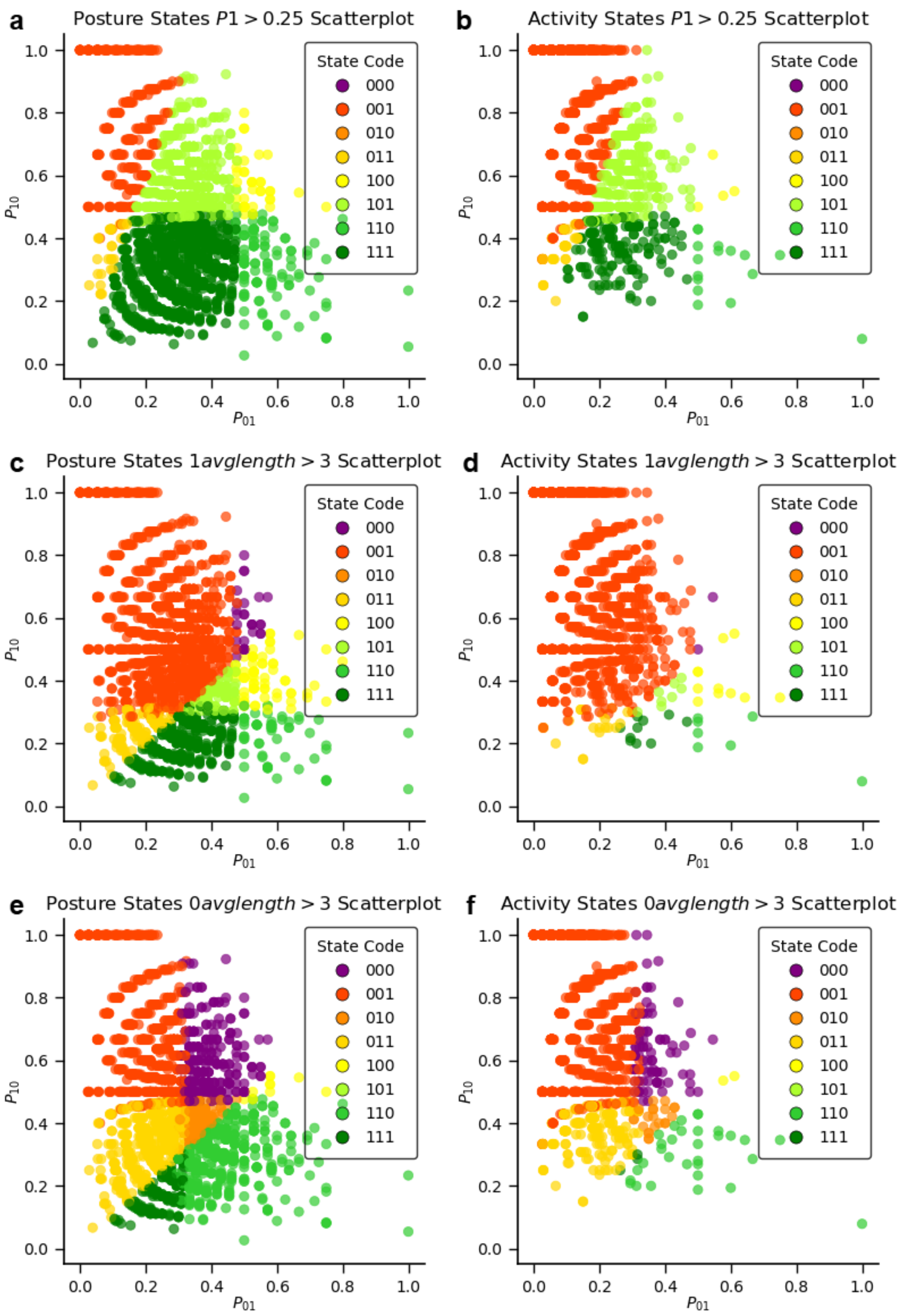

Supplementary Figure 3. Scatter Plots of $P_{01} - P_{10}$ Under Different Statistical Metric Thresholds

a,Presents the two-dimensional distribution of $P_{01} - P_{10}$ for all samples' posture time series. Each sample is assigned a three-digit binary state code based on the following criteria: the first digit is set to 1 if the sequence contains more than 25% "1"s; the second digit is 1 if the average run length of "1"s exceeds 2; the third digit is 1 if the average run length of "0"s exceeds 2. **b**, The $P_{01} - P_{10}$ distribution for activity time series, using the same state code assignment criteria as **a**. **c**, The first digit is set to 1 if the sequence contains more than 50% "1"s; the second digit is 1 if the average run length of "1"s exceeds 3; the third digit is 1 if the average run length of "0"s exceeds 2. **d**, The $P_{01} - P_{10}$ distribution for activity time series, using the same state code assignment criteria as **c**. **e**, the first digit is set to 1 if the sequence contains more than 50% "1"s; the second digit is 1 if the average run length of "1"s exceeds 2; the third digit is 1 if the average run length of "0"s exceeds 3. **f** The $P_{01} - P_{10}$ distribution for activity time series, using the same state code assignment criteria as **e**. All panels are based on $n = 2874$ biologically independent patient samples.

Table S4. LDA Evaluation for $P_{01} - P_{10}$ Scatter Plots Under Different Thresholds

Panel	Classification Accuracy (%)	Separation Ratio
**a**	92.48	4.716
**b**	93.70	5.515
**d**	88.69	4.268
**c**	98.36	4.916
**e**	84.48	4.104
**f**	95.34	5.153

The table presents the classification accuracy and separation ratios for Scatter Plots of $P_{01} - P_{10}$ Under Different Statistical Metric Thresholds. **a**,Posture time series, threshold: >25% 1's, avg run 1's >2, avg run 0's >2. **a**, Activity time series, same criteria as **a**. **c** Posture time series, threshold: >50% 1's, avg run 1's >3, avg run 0's >2. **d**, Activity time series, same criteria as textbfc. **e**, Posture time series, threshold: >50% 1's, avg run 1's >2, avg run 0's >3. **f**,Activity time series, same criteria as **e**.

Supplementary Note 6: MSE performance of FCNN, LSTM, and LightGBM

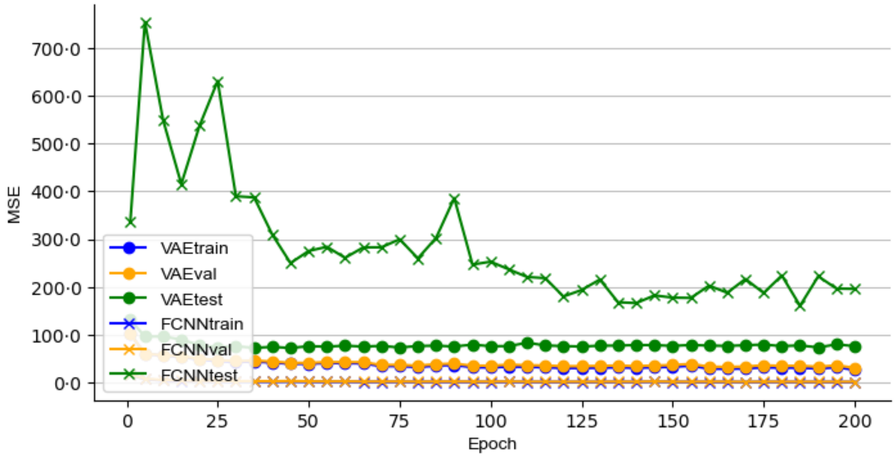

Supplementary Figure 4. MSE of Nocturnal Hypertension Prediction for ABPM-VAE and FCNN Models Across Training, Validation, and Test Sets

The figure displays the performance of the ABPM-VAE and FCNN models on ABPM subsets for predicting nocturnal hypertension. The blue, orange, and green circle lines represent the training, validation, and testing MSE values of the ABPM-VAE model, respectively. Similarly, the blue, orange, and green cross lines represent the corresponding MSE values for the LSTM model. The line plot shows how model error evolves over the training epochs.

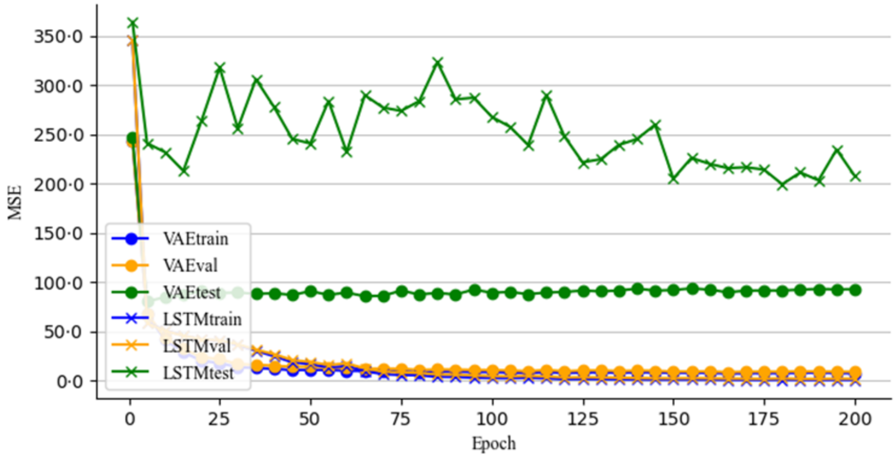

Supplementary Figure 5. MSE of Nocturnal Hypertension Prediction for ABPM-VAE and LSTM Models Across Training, Validation, and Test Sets

The figure displays the performance of the ABPM-VAE and LSTM models on ABPM subsets for predicting nocturnal hypertension. The blue, orange, and green circle lines represent the training, validation, and testing MSE values of the ABPM-VAE model, respectively. Similarly, the blue, orange, and green cross lines represent the corresponding MSE values for the LSTM model. The line plot shows how model error evolves over the training epochs.

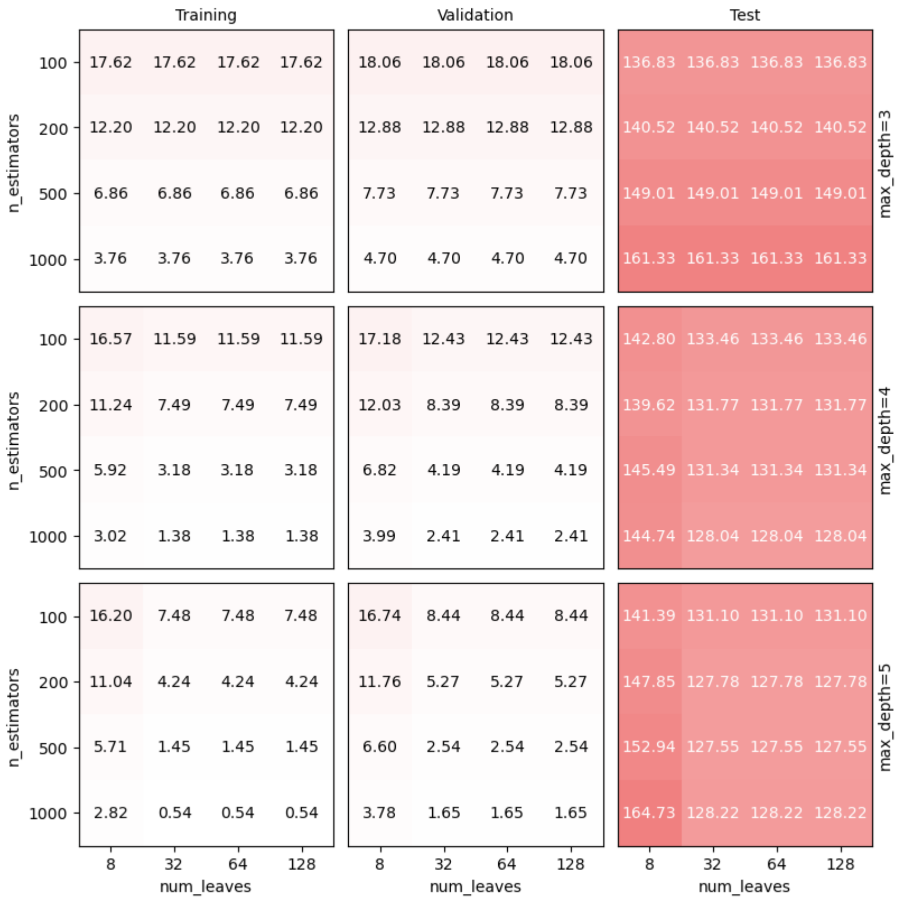

Supplementary Figure 6. Heatmaps of Average MSE Across Different LightGBM Hyperparameter Combinations

The figure displays the heatmaps of average MSE for the training, validation, and test sets under different LightGBM configurations. Each row corresponds to a fixed `max_depth` value, while `n_estimators` and `num_leaves` are shown along the vertical and horizontal axes, respectively. All heatmaps share the same color scale from white to light red, indicating increasing MSE values. Numerical annotations in each cell indicate the exact average MSE.

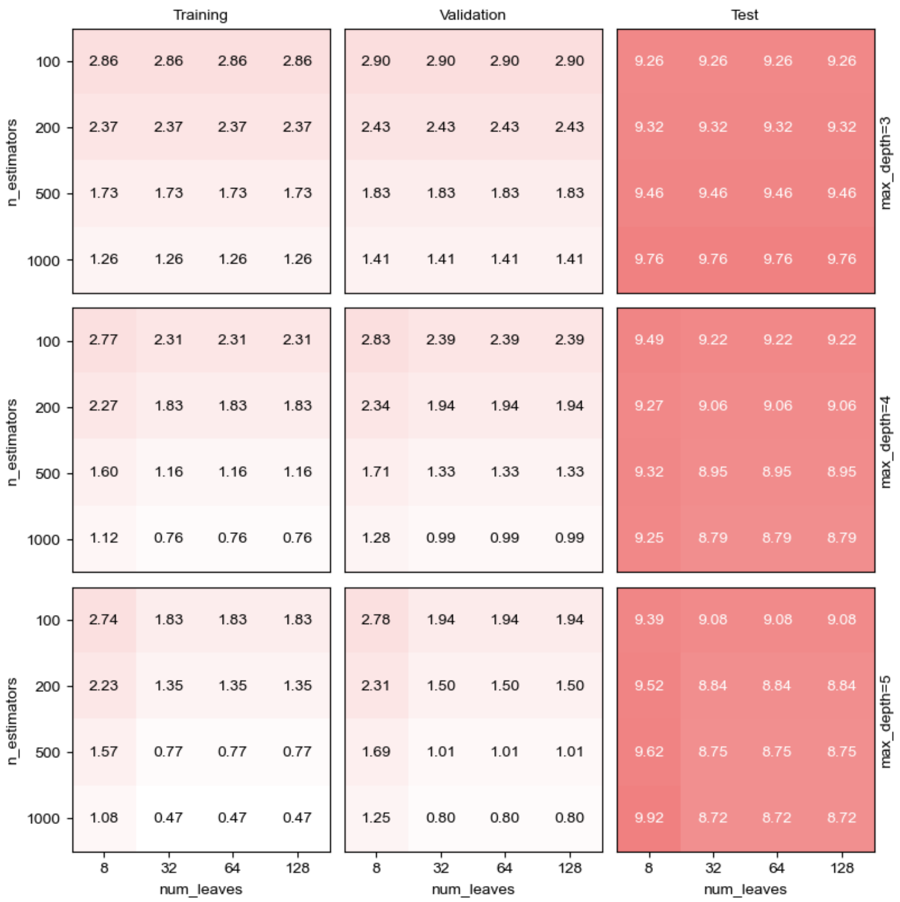

Supplementary Figure 7. Heatmaps of Average MAE Across Different LightGBM Hyperparameter Combinations

The figure displays the heatmaps of average MAE for the training, validation, and test sets under different LightGBM configurations. Each row corresponds to a fixed `max_depth` value, while `n_estimators` and `num_leaves` are shown along the vertical and horizontal axes, respectively. All heatmaps share the same color scale from white to light red, indicating increasing MAE values. Numerical annotations in each cell indicate the exact average MSE.

Supplementary Note 7: RMSE and MAE Performance of ABPM-VAE, Ablation, and Baseline Models across Training, Validation, and Test Data Sets

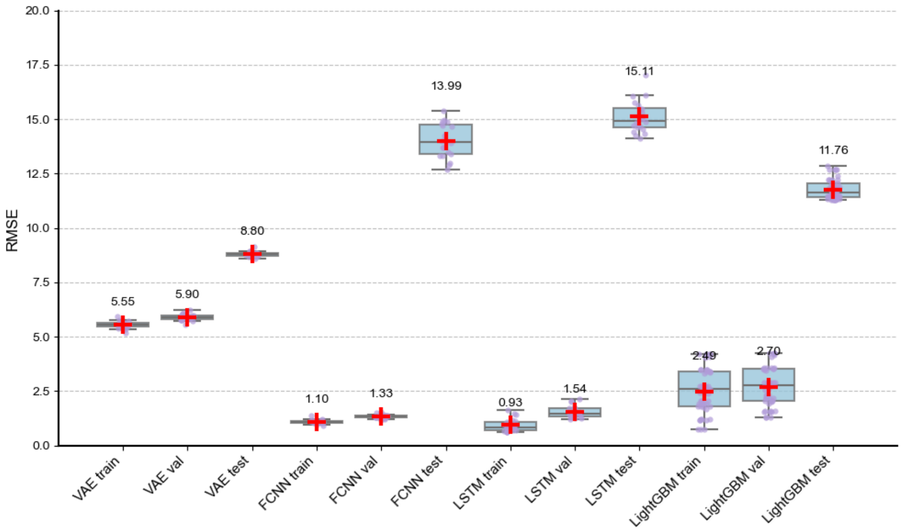

Supplementary Figure 8. RMSE Performance across Training, Validation, and Test Data Sets

All boxes are rendered in a uniform light blue hue. The centre line of each box denotes the median, while a red plus sign marks the mean; the mean value is annotated immediately above each box. The upper and lower edges of each box correspond to the first and third quartiles, respectively, and the whiskers extend to ± 1.5 times the interquartile range. Light purple dots represent the performance of each method under different test epochs or parameter settings. The ABPM-VAE model achieved the lowest RMSE of 8.80 (SD 0.11), significantly outperforming all other models ($p < 0.001$, two-tailed t-test). In comparison, the LightGBM model yielded an RMSE of 11.76 (SD 0.45), the FCNN ablation model 13.99 (SD 0.79), and the LSTM model 15.11 (SD 0.74).

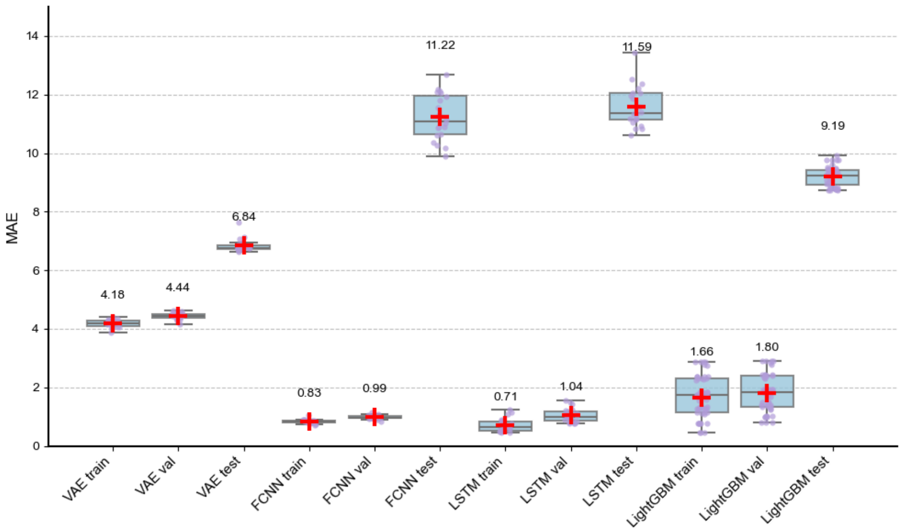

Supplementary Figure 9. MAE Performance across Training, Validation, and Test Data Sets

All boxes are rendered in a uniform light blue hue. The centre line of each box denotes the median, while a red plus sign marks the mean; the mean value is annotated immediately above each box. The upper and lower edges of each box correspond to the first and third quartiles, respectively, and the whiskers extend to ± 1.5 times the interquartile range. Light purple dots represent the performance of each method under different test epochs or parameter settings. The ABPM-VAE model achieved the lowest MAE of 6.48 (SD 0.23), significantly outperforming all other models ($p < 0.001$, two-tailed t-test). In comparison, the LightGBM model yielded an MAE of 9.19 (SD 0.33), the FCNN ablation model 11.22 (SD 0.79), and the LSTM model 11.59 (SD 0.70).

Supplementary Note 8: Classification Agreement Across Different Daytime Reading Selection Strategies

Table S5. Cohen's Kappa Analysis of Classification Agreement Across Different Daytime Reading Selection Strategies

Model	Prediction Target	Model Pair	Agreement Rate	Kappa (Evaluation)
FCNN	SBP	first vs last	83.10%	0.656 (Substantial)
FCNN	SBP	first vs alt_first	91.38%	0.824 (Almost Perfect)
FCNN	SBP	first vs alt_last	84.48%	0.686 (Substantial)
FCNN	SBP	last vs alt_first	84.83%	0.690 (Substantial)
FCNN	SBP	last vs alt_last	88.97%	0.777 (Substantial)
FCNN	SBP	alt_last vs alt_first	82.07%	0.636 (Substantial)
FCNN	DBP	first vs last	94.14%	0.721 (Substantial)
FCNN	DBP	first vs alt_first	97.59%	0.890 (Almost Perfect)
FCNN	DBP	first vs alt_last	97.93%	0.905 (Almost Perfect)
FCNN	DBP	last vs alt_first	95.17%	0.767 (Substantial)
FCNN	DBP	last vs alt_last	96.21%	0.814 (Almost Perfect)
FCNN	DBP	alt_last vs alt_first	95.52%	0.791 (Substantial)
ABPM-VAE	SBP	first vs last	88.28%	0.762 (Substantial)
ABPM-VAE	SBP	first vs alt_first	91.38%	0.825 (Almost Perfect)
ABPM-VAE	SBP	first vs alt_last	91.03%	0.818 (Almost Perfect)
ABPM-VAE	SBP	last vs alt_last	90.34%	0.804 (Almost Perfect)
ABPM-VAE	SBP	last vs alt_first	90.00%	0.798 (Substantial)
ABPM-VAE	SBP	alt_last vs alt_first	87.24%	0.741 (Substantial)
ABPM-VAE	DBP	first vs last	87.59%	0.745 (Substantial)
ABPM-VAE	DBP	first vs alt_last	88.62%	0.767 (Substantial)
ABPM-VAE	DBP	first vs alt_first	91.03%	0.815 (Almost Perfect)
ABPM-VAE	DBP	last vs alt_first	88.97%	0.772 (Substantial)
ABPM-VAE	DBP	last vs alt_last	90.69%	0.809 (Almost Perfect)
ABPM-VAE	DBP	alt_last vs alt_first	85.17%	0.696 (Substantial)

The table presents pairwise classification agreement rates and Cohen's Kappa coefficients for nocturnal hypertension predictions (separately for SBP and DBP) using four deterministic within-hour selection strategies in the FCNN and ABPM-VAE models: first (select the first measurement record within each hour), last (select the last measurement record within each hour), alt_first (for odd-numbered selections, select the first record in the hour; for even-numbered selections, select the last record in the hour), and alt_last (for odd-numbered selections, select the last record in the hour; for even-numbered selections, select the first record in the hour). Agreement Rate represents the percentage of consistent classifications between the two compared strategies; Kappa is Cohen's Kappa coefficient; Evaluation indicates the level of agreement according to the conventional standards proposed by Landis and Koch (1977) ($<$ 0.00: Poor; 0.00–0.20: Slight; 0.21–0.40: Fair; 0.41–0.60: Moderate; 0.61–0.80: Substantial; 0.81–1.00: Almost Perfect). All values are derived from pairwise comparisons among the four selection strategies.

Supplementary Note 9: Classifications of Dipping-Pattern vs Nocturnal Hypertension

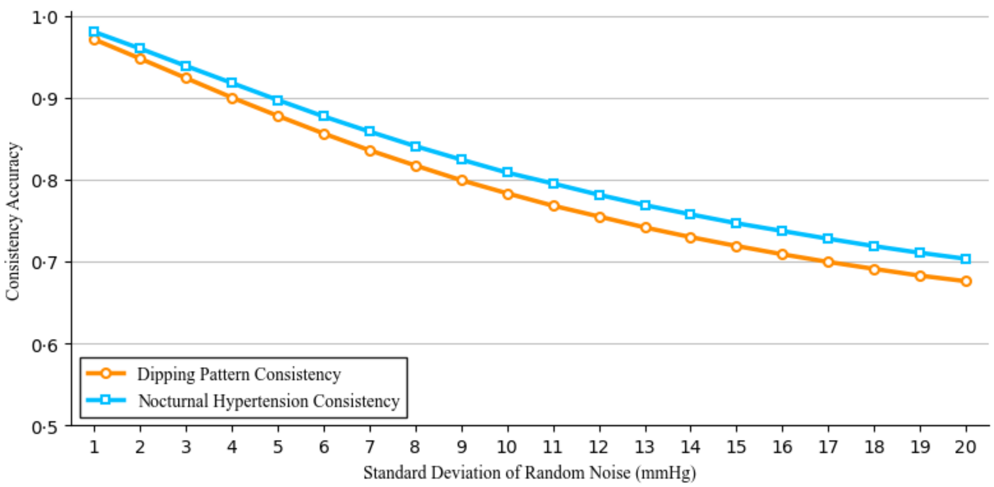

Supplementary Figure 10. Consistency accuracy for Dipping Patterns and Nocturnal Hypertension Classification versus Noise Standard Deviation (SD = 1—20 mmHg, 1000 repetitions).

For each patient in the test set, 1 000 Monte Carlo simulations were performed by adding signed absolute Gaussian noise $\pm|\mathcal{N}(0,\sigma)|$ to the true nocturnal SBP. Consistency accuracy represents the proportion of simulations in which the noisy classification matched the true label. The dipping pattern is markedly more sensitive to measurement noise than nocturnal hypertension.

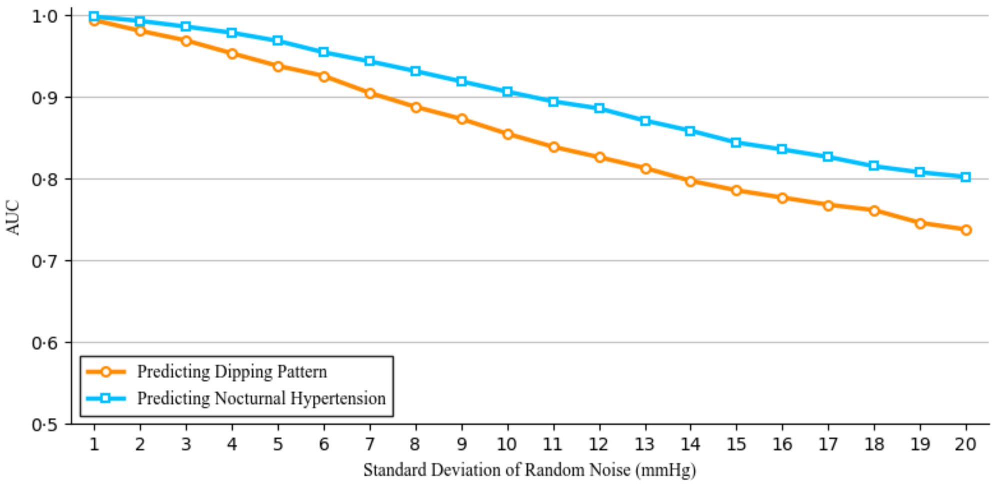

Supplementary Figure 11. Accuracy Curves for Dipping Patterns and Nocturnal Hypertension Classification versus Noise Standard Deviation (SD = 1—20 mmHg, 1000 repetitions).

For each patient in the test set and each noise level, 1 000 independent replications were performed. In each replication, nocturnal SBP was perturbed by adding signed absolute Gaussian noise $\pm|\mathcal{N}(0,\sigma)|$ with a fixed direction per patient and 10 Monte Carlo draws per replication; the predicted probability was the proportion of the 10 draws meeting the respective criterion. Points represent the mean of the AUC across the 1000 replications.

Table S6. Consistency accuracy for Dipping Patterns and Nocturnal Hypertension Classification versus Noise Standard Deviation (SD = 1—20 mmHg, 1000 repetitions).

Noise SD (mmHg)	Dipping Consistency	Nocturnal Consistency	Difference
1	0.971 (0.000)	0.980 (0.000)	-0.009 (0.000)
2	0.948 (0.000)	0.960 (0.000)	-0.012 (0.000)
3	0.924 (0.000)	0.939 (0.001)	-0.015 (0.001)
4	0.900 (0.001)	0.918 (0.000)	-0.018 (0.001)
5	0.878 (0.001)	0.897 (0.000)	-0.019 (0.001)
6	0.856 (0.000)	0.877 (0.001)	-0.021 (0.001)
7	0.836 (0.001)	0.859 (0.000)	-0.023 (0.001)
8	0.817 (0.000)	0.841 (0.000)	-0.024 (0.001)
9	0.799 (0.001)	0.824 (0.000)	-0.025 (0.001)
10	0.783 (0.000)	0.809 (0.000)	-0.025 (0.001)
11	0.768 (0.001)	0.795 (0.001)	-0.027 (0.001)
12	0.755 (0.000)	0.782 (0.001)	-0.026 (0.001)
13	0.742 (0.000)	0.769 (0.001)	-0.027 (0.001)
14	0.730 (0.001)	0.758 (0.001)	-0.028 (0.001)
15	0.719 (0.001)	0.747 (0.001)	-0.028 (0.001)
16	0.709 (0.000)	0.737 (0.001)	-0.028 (0.001)
17	0.700 (0.001)	0.728 (0.001)	-0.028 (0.000)
18	0.691 (0.001)	0.719 (0.001)	-0.028 (0.001)
19	0.683 (0.001)	0.711 (0.001)	-0.028 (0.001)
20	0.676 (0.001)	0.703 (0.001)	-0.027 (0.001)

Values are presented as mean (standard deviation).

Table S7. AUC for Dipping Patterns and Nocturnal Hypertension Classification versus Noise Standard Deviation (SD = 1—20 mmHg, 1000 repetitions).

Noise SD (mmHg)	Dipping AUC	Nocturnal AUC	Difference
1	0.994 (0.004)	0.999 (0.001)	-0.004 (0.004)
2	0.981 (0.009)	0.993 (0.004)	-0.012 (0.009)
3	0.969 (0.011)	0.986 (0.006)	-0.017 (0.013)
4	0.954 (0.011)	0.979 (0.007)	-0.025 (0.013)
5	0.938 (0.013)	0.969 (0.008)	-0.030 (0.015)
6	0.926 (0.016)	0.955 (0.009)	-0.029 (0.019)
7	0.905 (0.019)	0.944 (0.010)	-0.038 (0.020)
8	0.888 (0.020)	0.932 (0.011)	-0.044 (0.023)
9	0.873 (0.023)	0.919 (0.014)	-0.046 (0.028)
10	0.855 (0.018)	0.907 (0.016)	-0.051 (0.023)
11	0.839 (0.025)	0.895 (0.016)	-0.056 (0.028)
12	0.827 (0.028)	0.886 (0.016)	-0.060 (0.033)
13	0.813 (0.026)	0.871 (0.016)	-0.058 (0.030)
14	0.798 (0.029)	0.859 (0.019)	-0.061 (0.030)
15	0.786 (0.034)	0.845 (0.020)	-0.058 (0.034)
16	0.777 (0.026)	0.836 (0.021)	-0.059 (0.028)
17	0.768 (0.029)	0.827 (0.022)	-0.058 (0.034)
18	0.762 (0.036)	0.816 (0.022)	-0.054 (0.039)
19	0.746 (0.036)	0.808 (0.022)	-0.062 (0.040)
20	0.738 (0.030)	0.803 (0.023)	-0.064 (0.035)

Values are presented as mean (standard deviation).

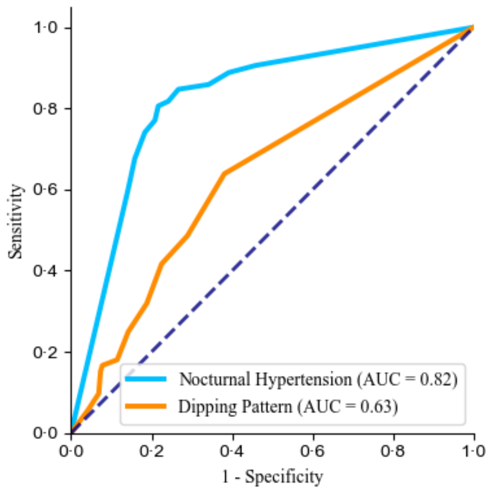

Supplementary Figure 12. ROC curves of ABPM-VAE for Nocturnal Hypertension and Dipping Pattern Prediction.

Supplementary Note 10: Analytical Estimation and Joint Least-Squares Fitting of Variance Increase After Filtering

Since the label is the joint BP-HR probability density map, BP variability can be directly computed from the label. For SBP variability (coefficient of variation, CV), we marginalize the joint map along the HR axis to obtain the marginal SBP distribution, compute the weighted mean and weighted SD using this marginal distribution as weights, and calculate CV as weighted SD divided by weighted mean. The same procedure is applied for DBP variability.

Since the coefficient of variation (CV) is defined as the standard deviation divided by the mean, the following analysis focuses on the standard deviation (SD) for simplicity.

The probability density contribution from a single raw SBP sample is modeled as a quasi-Gaussian kernel centered on that sample point, with bandwidth h of 0.630 (when $n = 16$ samples per time window) or 0.707 (when $n = 8$ samples per time window).

After applying the HR/BP physiological compatibility filter:

- The region to the right of the right blue dashed line is removed.
- The region to the left of the left red dashed line accounts for only 0.718% of the total probability mass. Moreover, because this removed left-tail region is closer to the population mean than the retained right-tail region, its contribution to the overall variance is even smaller ($<$ 0.718%). For the present quantitative estimation, this tiny left-tail contribution is therefore neglected.

Because the removed (slashed) regions and the symmetric red dots shown in Figure R1 are exactly symmetric with respect to the raw sample point, the difference in variance before and after filtering can be calculated analytically.

Let

$$\begin{aligned}
Var(\text{map}) &= \text{variance computed from the filtered probability density map,}\\
Var(\text{sample}) &= \text{variance computed from the original raw SBP measurements,}\\
y &= \text{distance between the individual's mean BP and the raw SBP measurement,}\\
P(x) &= \text{kernel density function (quasi-Gaussian) centered at the sample, with } x \text{ measured from the sample point,}\\
SD &= \text{standard deviation of the Gaussian kernel } P(x) \text{ used here, equal to } h \times y.
\end{aligned}$$

The variance increase introduced by a single kernel is

$$Var(\text{map}) - Var(\text{sample}) = \int_0^{2.448\,SD} P(x)\left[(y+x)^2 + (y-x)^2 - 2y^2\right] dx. \tag{S1}$$

This simplifies to

$$Var(\text{map}) - Var(\text{sample}) = 2\int_0^{2.448\,SD} P(x)\,x^2\,dx. \tag{S2}$$

Thus, the relative variance increase is

$$\frac{Var(\text{map}) - Var(\text{sample})}{Var(\text{sample})} = \frac{2\displaystyle\int_0^{2.448\,SD} P(x)\,x^2\,dx}{y^2}. \tag{S3}$$

Substituting $u = x/(hy)$ and simplifying yields

$$\frac{Var(\text{map}) - Var(\text{sample})}{Var(\text{sample})} = 2h^2\int_0^{2.448} P(u)\,u^2\,du. \tag{S4}$$

Therefore, when h is fixed, the final result is a constant independent of the value of y. The numerical evaluations are:

- When $n = 16$ (bandwidth $h = 0.630$): $\dfrac{Var(\text{map}) - Var(\text{sample})}{Var(\text{sample})} = 0.352$
- When $n = 8$ (bandwidth $h = 0.707$): $\dfrac{Var(\text{map}) - Var(\text{sample})}{Var(\text{sample})} = 0.444$

This leads to the following relationships:

$$SD(\text{sample}) = 0.859 \times SD(\text{map}) \qquad (n = 16,\ h = 0.630), \tag{S5}$$
$$SD(\text{sample}) = 0.832 \times SD(\text{map}) \qquad (n = 8,\ h = 0.707). \tag{S6}$$

Since dividing both sides by the mean BP leaves the ratio unchanged, the same coefficients apply directly to the coefficient of variation:

$$CV(\text{sample}) = 0.859 \times CV(\text{map}) \qquad (n = 16,\ h = 0.630), \tag{S7}$$
$$CV(\text{sample}) = 0.832 \times CV(\text{map}) \qquad (n = 8,\ h = 0.707). \tag{S8}$$

Since the theoretical derivation neglected the probability mass to the left of the left red dashed line (which accounts for only 0.718% of the total probability mass), the actual scaling coefficient is expected to be slightly higher than the theoretically derived value.

To obtain a more accurate empirical correction, we performed joint least-squares fitting of the optimal proportional coefficient k across all 12 subsets (stratified by daytime/nighttime and SBP/DBP). The resulting best-fit coefficients are:

- **Daytime:**
  - SBP: $k = 0.890$
  - DBP: $k = 0.894$
- **Nighttime:**
  - SBP: $k = 0.842$
  - DBP: $k = 0.843$

These empirical k values incorporate the small but non-negligible effect of the neglected left-tail probability mass and can be directly applied to adjust the relationship between $SD(\text{sample})/CV(\text{sample})$ and $SD(\text{map})/CV(\text{map})$.

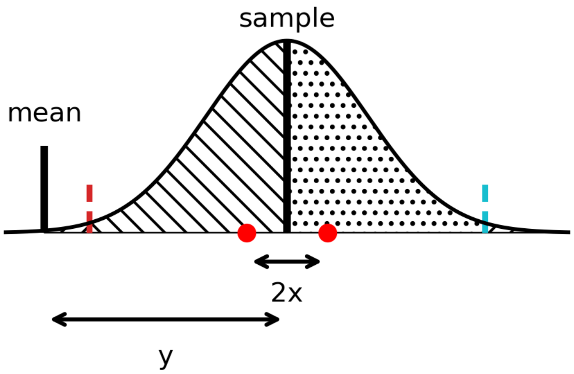

Supplementary Figure 13. Analytical Estimation Schematic Illustration.

"mean" denotes the mean BP; "sample" denotes a single raw SBP measurement; the distance between mean and sample is y. The left red dashed line and right blue dashed line indicate the locations where the kernel density reaches 5% of its maximum value on the left and right sides, respectively. The left and right red dots are symmetrically placed calculation points with respect to the "sample" point, and the distance between these two points is 2x.

Supplementary Note 11: Empirical Validation via Bland–Altman Analysis Across 12 Subsets

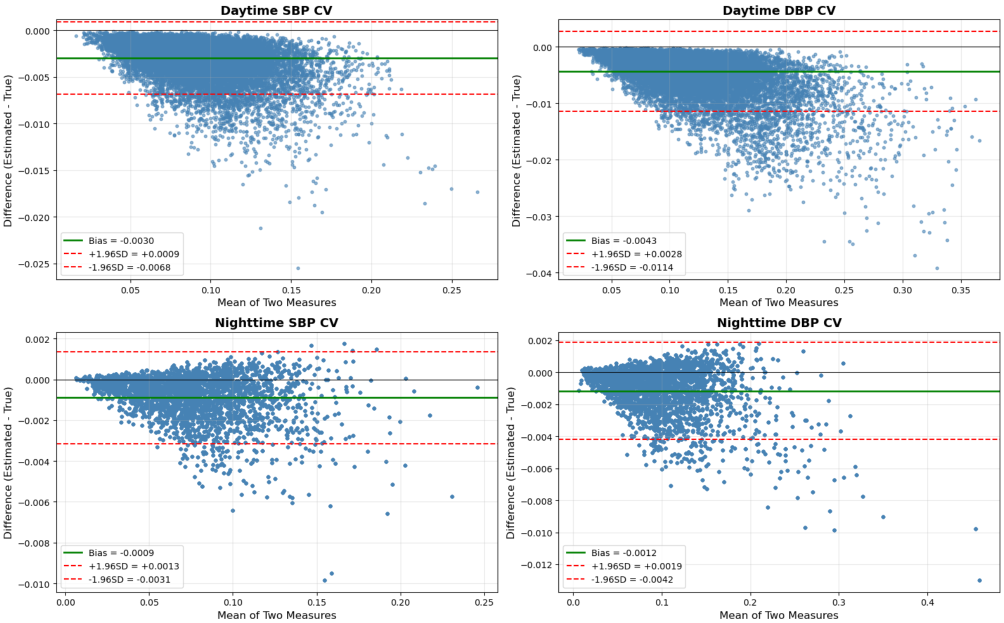

Supplementary Figure 14. Bland—Altman Analysis of CV Agreement in The Training Dataset (using theoretically derived scaling factors).

Bland–Altman plots evaluating agreement between the coefficient of variation derived from the filtered probability density map ($CV(\text{map})$) and the true coefficient of variation computed directly from raw BP measurements ($CV(\text{sample})$). The analysis includes 20,896 sub-segments from 2,612 biologically independent individuals in the training dataset. $CV(\text{map})$ was scaled using the theoretically derived factors: $CV(\text{sample}) = 0.859 \times CV(\text{map})$ (daytime) and $CV(\text{sample}) = 0.832 \times CV(\text{map})$ (nighttime). Each panel shows the difference (corrected $CV(\text{map}) - CV(\text{sample})$) versus the mean of the two measurements. The solid green line represents the mean bias, and the red dashed lines indicate the 95% limits of agreement (LoA). Agreement was excellent across all four conditions: daytime SBP CV: bias -0.30% (SD 0.20%), LoA -0.68% to 0.09%; daytime DBP CV: bias -0.43% (SD 0.36%), LoA -1.14% to $+0.28\%$; nighttime SBP CV: bias -0.09% (SD 0.11%), LoA -0.31% to 0.13%; nighttime DBP CV: bias -0.12% (SD 0.16%), LoA -0.42% to $+0.19\%$.

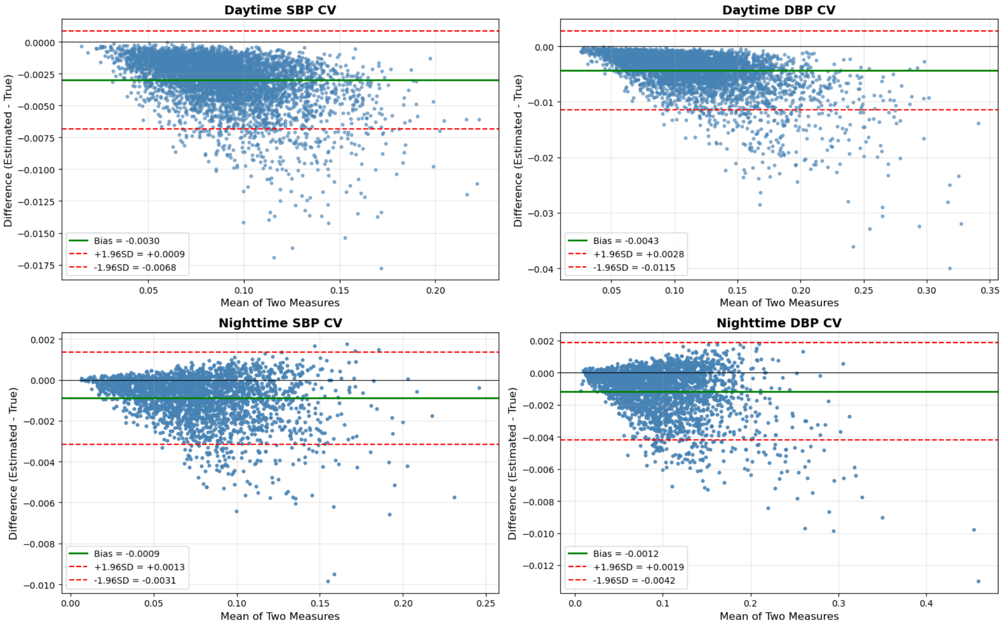

Supplementary Figure 15. Bland—Altman Analysis of CV Agreement in The Validation Dataset (using theoretically derived scaling factors).

Bland–Altman plots evaluating agreement between the coefficient of variation derived from the filtered probability density map (CV(map)) and the true coefficient of variation computed directly from raw BP measurements (CV(sample)) across 5,224 sub-segments generated from 2,612 biologically independent individuals in the validation dataset. CV(map) was multiplied by the theoretically derived scaling factors: $CV(\text{sample}) = 0.859 \times CV(\text{map})$ (daytime) and $CV(\text{sample}) = 0.832 \times CV(\text{map})$ (nighttime). Each panel displays the difference (corrected $CV(\text{map}) - CV(\text{sample})$) versus the mean of the two measurements, with the solid green line indicating mean bias and red dashed lines denoting the 95% limits of agreement (LoA). Agreement was excellent in all four conditions: daytime SBP CV: bias -0.30% (SD 0.20%), LoA -0.68% to 0.09%; daytime DBP CV: bias -0.43% (SD 0.36%), LoA -1.15% to $+0.28\%$; nighttime SBP CV: bias -0.09% (SD 0.11%), LoA -0.31% to 0.13%; nighttime DBP CV: bias -0.12% (SD 0.16%), LoA -0.42% to $+0.19\%$.

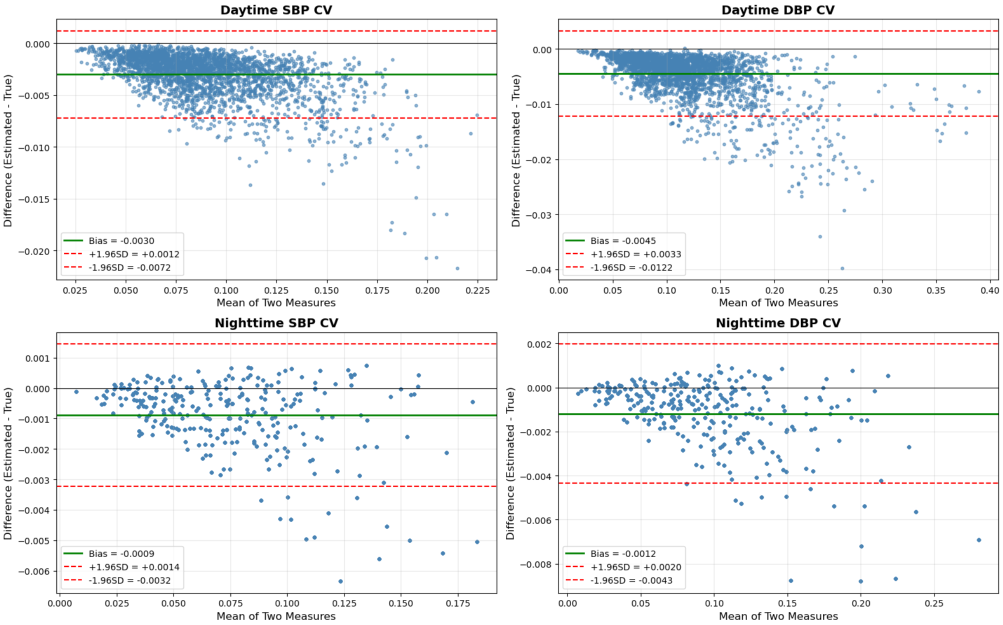

Supplementary Figure 16. Bland—Altman Analysis of CV Agreement in The Testing Dataset (using theoretically derived scaling factors).

Bland–Altman plots evaluating agreement between the coefficient of variation derived from the filtered probability density map (CV(map)) and the true coefficient of variation computed directly from raw BP measurements (CV(sample)) across 2,900 sub-segments generated from 290 biologically independent individuals in the testing dataset. CV(map) was multiplied by the theoretically derived scaling factors: $CV(\text{sample}) = 0.859 \times CV(\text{map})$ (daytime) and $CV(\text{sample}) = 0.832 \times CV(\text{map})$ (nighttime). Each panel displays the difference (corrected $CV(\text{map}) - CV(\text{sample})$) versus the mean of the two measurements, with the solid green line indicating mean bias and red dashed lines denoting the 95% limits of agreement (LoA). Agreement was excellent in all four conditions: daytime SBP CV: bias -0.30% (SD 0.21%), LoA -0.68% to 0.09%; daytime DBP CV: bias -0.45% (SD 0.39%), LoA -1.22% to $+0.33\%$; nighttime SBP CV: bias -0.09% (SD 0.12%), LoA -0.32% to 0.14%; nighttime DBP CV: bias -0.12% (SD 0.16%), LoA -0.43% to $+0.20\%$.

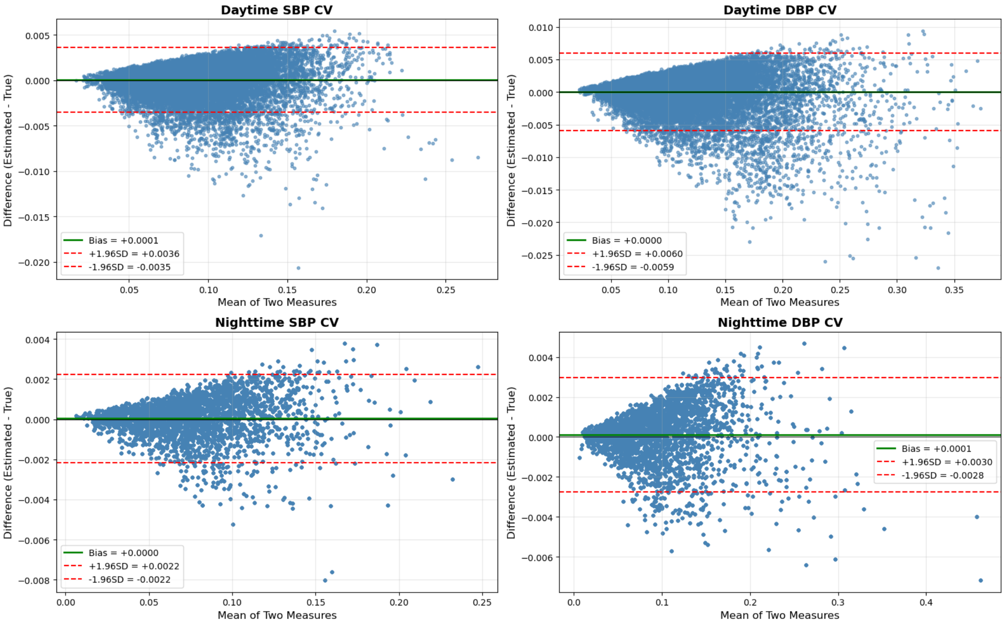

Supplementary Figure 17. Bland—Altman Analysis of CV Agreement in The Training Dataset (using empirically optimized scaling factors).

Bland–Altman plots evaluating agreement between the coefficient of variation derived from the filtered probability density map (CV(map)) and the true coefficient of variation computed directly from raw BP measurements (CV(sample)) across 20,896 sub-segments generated from 2,612 biologically independent individuals in the training dataset. CV(map) was multiplied by the empirically optimized scaling factors obtained by joint least-squares fitting across all subsets: daytime SBP $k = 0.890$, daytime DBP $k = 0.894$, nighttime SBP $k = 0.842$, nighttime DBP $k = 0.843$. Each panel displays the difference (corrected $CV(\text{map}) - CV(\text{sample})$) versus the mean of the two measurements, with the solid green line indicating mean bias and red dashed lines denoting the 95% limits of agreement (LoA). Agreement was excellent in all four conditions: daytime SBP CV: bias 0.01% (SD 0.18%), LoA -0.35% to 0.36%; daytime DBP CV: bias 0.00% (SD 0.30%), LoA -0.59% to $+0.60\%$; nighttime SBP CV: bias 0.00% (SD 0.11%), LoA -0.22% to 0.22%; nighttime DBP CV: bias 0.01% (SD 0.15%), LoA -0.28% to $+0.30\%$.

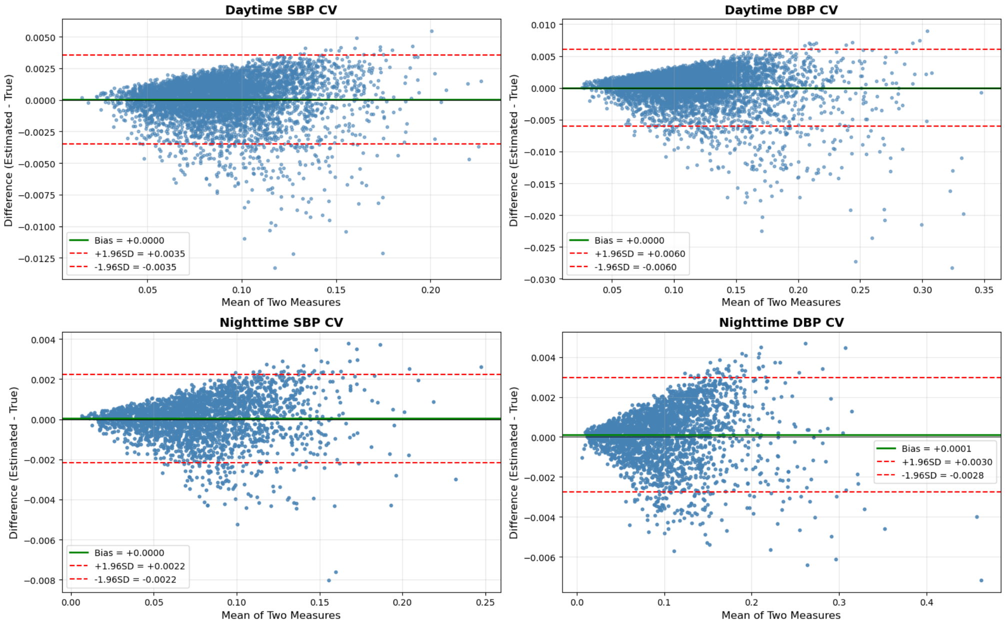

Supplementary Figure 18. Bland—Altman Analysis of CV Agreement in The Validation Dataset (using empirically optimized scaling factors).

Bland–Altman plots evaluating agreement between the coefficient of variation derived from the filtered probability density map (CV(map)) and the true coefficient of variation computed directly from raw BP measurements (CV(sample)) across 5,224 sub-segments generated from 2,612 biologically independent individuals in the validation dataset. CV(map) was multiplied by the empirically optimized scaling factors obtained by joint least-squares fitting across all subsets: daytime SBP $k = 0.890$, daytime DBP $k = 0.894$, nighttime SBP $k = 0.842$, nighttime DBP $k = 0.843$. Each panel displays the difference (corrected $CV(\text{map}) - CV(\text{sample})$) versus the mean of the two measurements, with the solid green line indicating mean bias and red dashed lines denoting the 95% limits of agreement (LoA). Agreement was excellent in all four conditions: daytime SBP CV: bias 0.01% (SD 0.18%), LoA −0.35% to 0.35%; daytime DBP CV: bias 0.00% (SD 0.31%), LoA −0.60% to +0.60%; nighttime SBP CV: bias 0.00% (SD 0.11%), LoA −0.22% to 0.22%; nighttime DBP CV: bias 0.00% (SD 0.15%), LoA −0.28% to +0.30%.

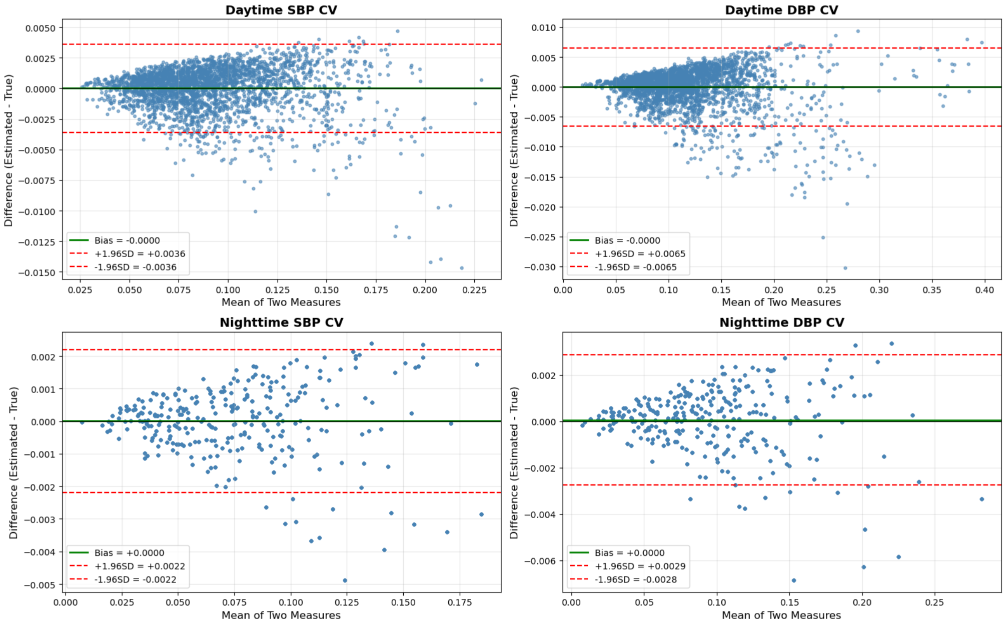

Supplementary Figure 19. Bland—Altman Analysis of CV Agreement in The Validation Dataset (using empirically optimized scaling factors).

Bland–Altman plots evaluating agreement between the coefficient of variation derived from the filtered probability density map (CV(map)) and the true coefficient of variation computed directly from raw BP measurements (CV(sample)) across 2,900 sub-segments generated from 290 biologically independent individuals in the validation dataset. CV(map) was multiplied by the empirically optimized scaling factors obtained by joint least-squares fitting across all subsets: daytime SBP $k = 0.890$, daytime DBP $k = 0.894$, nighttime SBP $k = 0.842$, nighttime DBP $k = 0.843$. Each panel displays the difference (corrected $CV(\text{map}) - CV(\text{sample})$) versus the mean of the two measurements, with the solid green line indicating mean bias and red dashed lines denoting the 95% limits of agreement (LoA). Agreement was excellent in all four conditions: daytime SBP CV: bias 0.01% (SD 0.18%), LoA -0.36% to 0.36%; daytime DBP CV: bias 0.00% (SD 0.33%), LoA -0.65% to $+0.65\%$; nighttime SBP CV: bias 0.00% (SD 0.11%), LoA -0.22% to 0.22%; nighttime DBP CV: bias 0.00% (SD 0.14%), LoA -0.28% to $+0.29\%$.

Table S8. Bland–Altman Analysis of CV Agreement Across Training, Validation, and Testing Datasets(using theoretically derived scaling factors).

Dataset	CV Type	n	Bias (%)	SD (%)	LoA lower (%)	LoA upper (%)	Within LoA (%)
VAEtrain	Daytime SBP CV	20896	-0.30	0.20	-0.68	0.09	95.19
VAEtrain	Daytime DBP CV	20896	-0.43	0.36	-1.14	0.28	95.02
VAEtrain	Nighttime SBP CV	20896	-0.09	0.11	-0.31	0.13	95.37
VAEtrain	Nighttime DBP CV	20896	-0.12	0.16	-0.42	0.19	94.79
VAEval	Daytime SBP CV	5224	-0.30	0.20	-0.68	0.09	95.25
VAEval	Daytime DBP CV	5224	-0.43	0.36	-1.15	0.28	95.35
VAEval	Nighttime SBP CV	5224	-0.09	0.11	-0.31	0.13	95.37
VAEval	Nighttime DBP CV	5224	-0.12	0.16	-0.42	0.19	94.79
VAEtest	Daytime SBP CV	2900	-0.30	0.21	-0.72	0.12	95.38
VAEtest	Daytime DBP CV	2900	-0.45	0.39	-1.22	0.33	94.90
VAEtest	Nighttime SBP CV	2900	-0.09	0.12	-0.32	0.14	95.17
VAEtest	Nighttime DBP CV	2900	-0.12	0.16	-0.43	0.20	95.17

Bland–Altman agreement parameters between the map-derived coefficient of variation (CV(map), corrected by theoretical scaling factors) and the true coefficient of variation calculated directly from raw measurements (CV(sample)) for SBP and DBP during daytime and nighttime periods. Bias represents the mean difference (corrected CV(map) $-$ CV(sample)); SD is the standard deviation of the differences; 95% limits of agreement (LoA) are calculated as bias $\pm 1.96\times$ SD; Points within LoA (%) indicates the percentage of data points falling within the 95% LoA. All values are expressed in percentage units. CV(map) was corrected prior to analysis using the theoretical scaling factors 0.859 (daytime) and 0.832 (nighttime).

Table S9. Bland–Altman Analysis of CV Agreement Across Training, Validation, and Testing Datasets(using empirically optimized scaling factors derived scaling factors).

Dataset	CV Type	n	Bias (%)	SD (%)	LoA lower (%)	LoA upper (%)	Within LoA (%)
VAEtrain	Daytime SBP CV	20896	0.01	0.18	-0.35	0.36	95.22
VAEtrain	Daytime DBP CV	20896	0.00	0.30	-0.59	0.60	95.14
VAEtrain	Nighttime SBP CV	20896	0.00	0.11	-0.22	0.22	94.79
VAEtrain	Nighttime DBP CV	20896	0.01	0.15	-0.28	0.30	94.18
VAEval	Daytime SBP CV	5224	0.00	0.18	-0.35	0.35	95.33
VAEval	Daytime DBP CV	5224	0.00	0.31	-0.60	0.60	95.25
VAEval	Nighttime SBP CV	5224	0.00	0.11	-0.22	0.22	94.79
VAEval	Nighttime DBP CV	5224	0.01	0.15	-0.28	0.30	94.18
VAEtest	Daytime SBP CV	2900	0.00	0.18	-0.36	0.36	95.48
VAEtest	Daytime DBP CV	2900	0.00	0.33	-0.65	0.65	95.00
VAEtest	Nighttime SBP CV	2900	0.00	0.11	-0.22	0.22	94.83
VAEtest	Nighttime DBP CV	2900	0.00	0.14	-0.28	0.29	95.17

Bland–Altman agreement parameters between the map-derived coefficient of variation (CV(map), corrected by empirically optimized scaling factors) and the true coefficient of variation calculated directly from raw measurements (CV(sample)) for SBP and DBP during daytime and nighttime periods. Bias represents the mean difference (corrected CV(map) − CV(sample)); SD is the standard deviation of the differences; 95% limits of agreement (LoA) are calculated as bias $\pm 1.96\times$ SD; Points within LoA (%) indicates the percentage of data points falling within the 95% LoA. All values are expressed in percentage units. CV(map) was corrected prior to analysis using the optimized scaling factors obtained by joint least-squares fitting: daytime SBP CV $k = 0.890$; daytime DBP CV $k = 0.894$; nighttime SBP CV $k = 0.842$; nighttime DBP CV $k = 0.843$.

Table S10. Evaluation of ABPM-VAE Predicted BP Variability.

Parameter	Mean (%)	SD (%)
True SBP CV	8.9413	4.1379
True DBP CV	11.5009	5.7078
Adjusted Predicted SBP CV	12.2993	1.2635
Adjusted Predicted DBP CV	15.6086	4.2169
SBP CV MAE (true vs pred)	6.2785	3.4298
DBP CV MAE (true vs pred)	6.3730	4.2897
