# Supplementary material for: Deep learning prediction of nocturnal hypertension for patients intolerant to ambulatory blood pressure monitoring
Source: Commun Med (Lond). 2026 May 8;6:396. doi: 10.1038/s43856-026-01639-x (PMC13369173; doi:10.1038/s43856-026-01639-x)
Supplement: Supplementary file 3 — Description of Additional Supplementary files [file 43856_2026_1639_MOESM3_ESM.docx]

**Description of Additional Supplementary Files**

File name: Supplementary Data 1
Description: ABPM data of patient ID 1620#2021-03-20, Source data for Fig. 1

File name: Supplementary Data 2
Description: Transition probabilities p01 and p10 of activity for all samples, source data for Fig. 2 and Fig. 4

File name: Supplementary Data 3
Description: Transition probabilities p01 and p10 of posture for all samples, source data for Fig. 2 and Fig. 4

File name: Supplementary Data 4
Description: Prediction accuracy of nocturnal hypertension for ABPM-VAE and FCNN models across training, validation, and test sets, source data for Fig. 5

File name: Supplementary Data 5
Description: Test set results of ABPM-VAE model, source data for Fig. 6

File name: Supplementary Data 6
Description: Test set results of FCNN model, source data for Fig. 6

File name: Supplementary Data 7
Description: MSE performance of ABPM-VAE, ablation, and baseline Models across training, validation, and test Data Sets, source data for Fig. 7

File name: Supplementary Data 8
Description: Targeted perturbation result of test set when activity/posture transition probabilities fixed to 0101

File name: Supplementary Data 9
Description: Targeted perturbation result of test set when activity/posture transition probabilities fixed to 1010
