# Supplementary material for: Deep learning prediction of nocturnal hypertension for patients intolerant to ambulatory blood pressure monitoring
Source: Commun Med (Lond). 2026 May 8;6:396. doi: 10.1038/s43856-026-01639-x (PMC13369173; doi:10.1038/s43856-026-01639-x)
Supplement: Supplementary file 1 — Transparent Peer Review file [file 43856_2026_1639_MOESM1_ESM.pdf]

nature portfolio

Peer Review File

Deep learning prediction of nocturnal hypertension for patients intolerant to ambulatory blood pressure monitoring

Corresponding Author: Dr Yifan Lin

This file contains all reviewer reports in order by version, followed by all author rebuttals in order by version.

Version 0:

Reviewer comments:

Reviewer #1

(Remarks to the Author)
This study employs KDE-based entropy reduction combined with deep learning models to predict and classify nocturnal hypertension using ABPM data. However, the peer review process has revealed several critical issues:

1. While the proposed method meets practical engineering requirements, its overall contribution in terms of innovation is limited, particularly regarding the model architecture and feature processing techniques.

2. Although the study primarily focuses on classification, reporting only performance metrics such as AUC and PPV in the results section is insufficient. Incorporating regression analysis and providing direct error metrics between the actual and predicted blood pressure values (e.g., MSE, RMSE, MAE) would significantly enhance the reliability and robustness of the study's findings.

3. It is necessary to investigate how the characteristics of ABPM-recorded data influence the model's prediction process. Improving the interpretability of model features would further strengthen the study.

Reviewer #2

(Remarks to the Author)
The authors present an interesting framework on nocturnal hypertension diagnosis from limited ABPM data only from the day time. They use a KDE based pre-processing strategy that is then fed into a variational autoencoder (VAE) to predict the systolic and diastolic hypertension labels for the corresponding patient. The labels are fetched based on if the patients have over a 120 mmHg, and/or 70 mmHg mean systolic and diastolic BP during night time.
The motivation of using the day time data for nocturnal hypertension diagnosis is valid. However, there are major concerns with this study that I have identified;
1. One of the fundamental reason clinicians use nocturnal BP for hypertension diagnosis is due the limited external stimuli (e.g., noise in day time due to movement, physiological stress and stimuli bias) that is not occurrent during night time. If one uses day time BP, how would they guarantee that the data is bias/noise free?
2. The clinicians for nocturnal BP care about both the dipping patterns in BP (and potentially diagnose BP non-dippers) and the BP variability (a strong predictor of cardiovascular disorder and dysfunction). The proposed analysis only looks into two threshold values. The labels need to be increased to these phenomena for clinical utility for this work. Also, using a HR/BP filter may actually reduce the BP variability which is a very important measure.
3. A major concern I have is the ablation study (FCNN) that is used for benchmarking against the performance of the VAE model. I am quoting the authors here: " The FCNN model takes as input the timestamp, SBP, DBP, heart rate, activity state, and positional state, with nighttime systolic and diastolic BP values set to zero." Assigning zero values to nighttime systolic and diastolic BP values. This choice is problematic for two reasons. First, replacing nighttime BP with zeros does not reflect physiological reality, no blood pressure can be zero, and thus introduces an artificial and misleading signal into the data. The model is effectively encouraged to learn a systemically low BP during night time, which biases it toward underestimating nocturnal hypertension. Second, this "handicap" is not applied to to the ABPM-VAE, which receives a clean representation (KDE maps of daytime BP–HR) without the same zeroing of BP during night time. I recommend the authors to perform a more fair strategy. For example, why not restricting the FCNN inputs to daytime values only? Or, implement a proper masking mechanism (e.g., a missingness indicator) to denote withheld nighttime values. With the current approach,

that fills night time BP with zeros, I am concerned about the fairness of the ablation and the validty of the reported performance improvement with the proposed technique.
4. A minor consideration: the authors highlight a positive predictive value (PPV) of ~91% at threshold 0.6, but PPV is highly dependent on disease prevalence. In a population with lower prevalence, the PPV would be much lower even if sensitivity and specificity were unchanged. Please also report sensitivity, specificity, and NPV alongside PPV.

Reviewer #3

(Remarks to the Author)
The paper tackles a common but under-addressed problem: distribution mismatch between training and test data caused by outliers and measurement noise in ambulatory blood pressure monitoring (ABPM) and related physiological signals. The authors propose a strategy, using multi-signal inputs (e.g., BP, HR, body position) with an outlier-aware pipeline, to improve the prediction of nocturnal hypertension from 1D signals.
Novelty & interest: The focus on outlier-robust learning for nocturnal hypertension appears novel in this specific clinical context and will likely interest both the physiological signal processing community and clinicians working with ABPM.
Overall Idea: The core idea is intuitive and promising. With some clarifications, consistency checks, and targeted analyses (see below), the paper can be made more convincing and broadly useful.
Comments:
1. The paper states: “2,920 ABPM recordings from 2,876 patients (1,492 men [51.9%] and 1,382 women [48.1%]).” While the percentages sum to 100%, 1,492 + 1,382 = 2,874, not 2,876. Please reconcile the counts.
2. The authors select one reading per hour from 06:00–22:00 to yield 16 daytime readings. If the selection is random within each hour, adjacent readings could be temporally clustered across hours. Consider specifying a deterministic spacing rule or report sensitivity analyses showing that the model’s performance is insensitive to the within-hour selection.
3. The rationale for adding a noise vector in the latent space needs elaboration. What distribution is used? If you remove outliers from density maps while adding noise in latent space, please articulate the conceptual goal. Please add ABPM-VAE results without noise injection.
4. The manuscript oscillates between predicting nocturnal hypertension (a binary clinical endpoint) and producing BP-HR probability density maps (e.g., Fig. 4 title). If the model output is a 2D map but the reported endpoint is nocturnal hypertension, please make explicit how the 2D output is converted into the binary label and how the separate systolic and diastolic metrics are derived.
5. The text states that 16 daytime + 8 nighttime readings form a 24-hour subset yet later says only daytime ABPM is used to predict nocturnal hypertension. If nighttime data are used as labels (ground truth) and daytime as predictors, please state this and include a schematic of the data flow (inputs, labels).

Reviewer #4

(Remarks to the Author)
I co-reviewed this manuscript with one of the reviewers who provided the listed reports. This is part of the Communications Medicine initiative to facilitate training in peer review and to provide appropriate recognition for Early Career Researchers who co-review manuscripts.

Version 1:

Reviewer comments:

Reviewer #1

(Remarks to the Author)
This study addresses the important clinical issue of intolerance to nighttime ABPM and proposes an innovative KDE-VAE deep learning framework, which has methodological exploratory value. However, its evaluation framework suffers from fundamental deficiencies:

Blood pressure regression evaluation is the foundation. The model must first demonstrate its ability to accurately predict blood pressure values using metrics such as RMSE and MAE, and its performance should be benchmarked against AAMI/ESH standards. Classification is merely a derivative application based on regression results and is meaningful only if the regression performance meets acceptable standards.

In the revised manuscript, the authors overemphasize classification performance while avoiding proper validation of regression accuracy.

Moreover, the blood pressure prediction accuracy does not meet the AAMI/ESH clinical criteria.

Reviewer #2

(Remarks to the Author)
Thank you for your response letter and revisions. My comments are addressed comprehensively and systematically. I have no remaining concerns. Good luck.

Reviewer #3

(Remarks to the Author)
The authors have comprehensively responded to previous reviewer comments, performed extensive additional analyses, and added relevant discussion regarding the revised findings. The revised manuscript is comprehensive and provides a strong basis for further ML based hypertension phenotyping.

Version 2:

Reviewer comments:

Reviewer #1

(Remarks to the Author)
Although the comments have been addressed, the corresponding supplementary content (Supplementary Figures 8 and 9) is not included in the manuscript. Acceptance is recommended after revision.

Open Access This Peer Review File is licensed under a Creative Commons Attribution 4.0 International License, which permits use, sharing, adaptation, distribution and reproduction in any medium or format, as long as you give appropriate credit to the original author(s) and the source, provide a link to the Creative Commons license, and indicate if changes were made.
In cases where reviewers are anonymous, credit should be given to 'Anonymous Referee' and the source.
The images or other third party material in this Peer Review File are included in the article's Creative Commons license, unless indicated otherwise in a credit line to the material. If material is not included in the article's Creative Commons license and your intended use is not permitted by statutory regulation or exceeds the permitted use, you will need to obtain permission directly from the copyright holder.
To view a copy of this license, visit https://creativecommons.org/licenses/by/4.0/
